# Supplementary material for: Analysis of 11,430 recombinant protein production experiments reveals that protein yield is tunable by synonymous codon changes of translation initiation sites
Source: PLoS Comput Biol. 2021 Oct 5;17(10):e1009461. doi: 10.1371/journal.pcbi.1009461 (PMC8519471; doi:10.1371/journal.pcbi.1009461)
Supplement: S1 Fig — Green unfilled triangles indicate the regions before and after scaling (left and right panels, respectively). A: For E. coli, we used a representative GFP expression dataset from Cambray et al. (2018) [16]. The reporter library consists of GFP fused in-frame with a library of 96-nt upstream sequences (N = 14,425). B: For S. cerevisiae, we used a YFP expression dataset from Dvir et al. (2013) [19]. The YFP reporter library consists of 2,041 random decameric nucleotides inserted at the upstream of YFP start codon. C: For M. musculus, we used the GFP expression dataset from Noderer et al. (2014) [34]. The GFP reporter library consists of 65,536 random hexameric and dimeric nucleotides inserted at the upstream and downstream of GFP start codon, respectively. Rs, Spearman’s rho. (PDF) [file pcbi.1009461.s001.pdf]

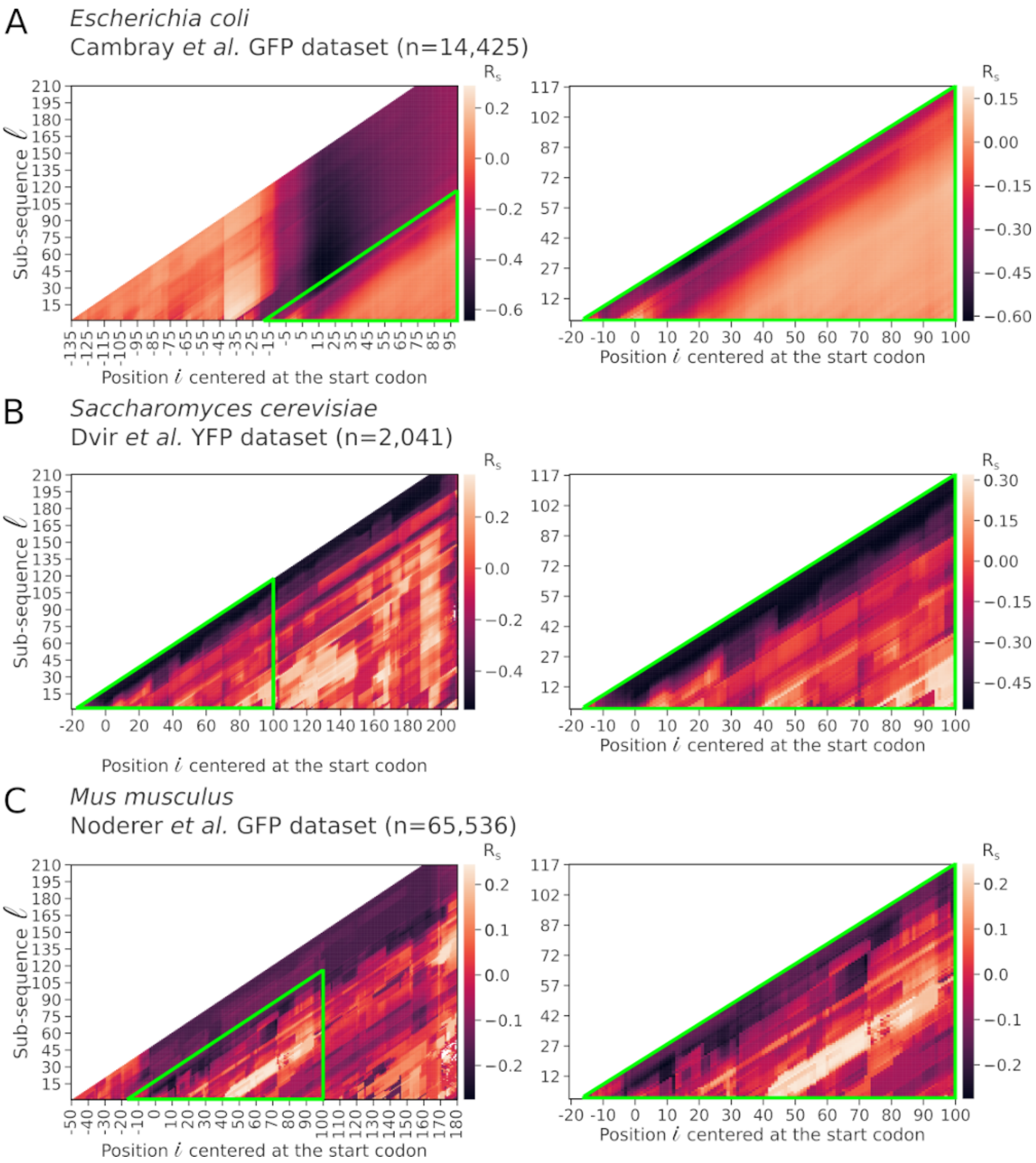
A
Escherichia coli
Cambray et al. GFP dataset (n=14,425)
B
Saccharomyces cerevisiae
Dvir et al. YFP dataset (n=2,041)
C
Mus musculus
Noderer et al. GFP dataset (n=65,536)
Sub-sequence ℓ
Position i centered at the start codon
Rs

S1 Fig
