# Supplementary figures and images for: Analysis of 11,430 recombinant protein production experiments reveals that protein yield is tunable by synonymous codon changes of translation initiation sites
Source: PLoS Comput Biol. 2021 Oct 5;17(10):e1009461. doi: 10.1371/journal.pcbi.1009461 (PMC8519471; doi:10.1371/journal.pcbi.1009461)

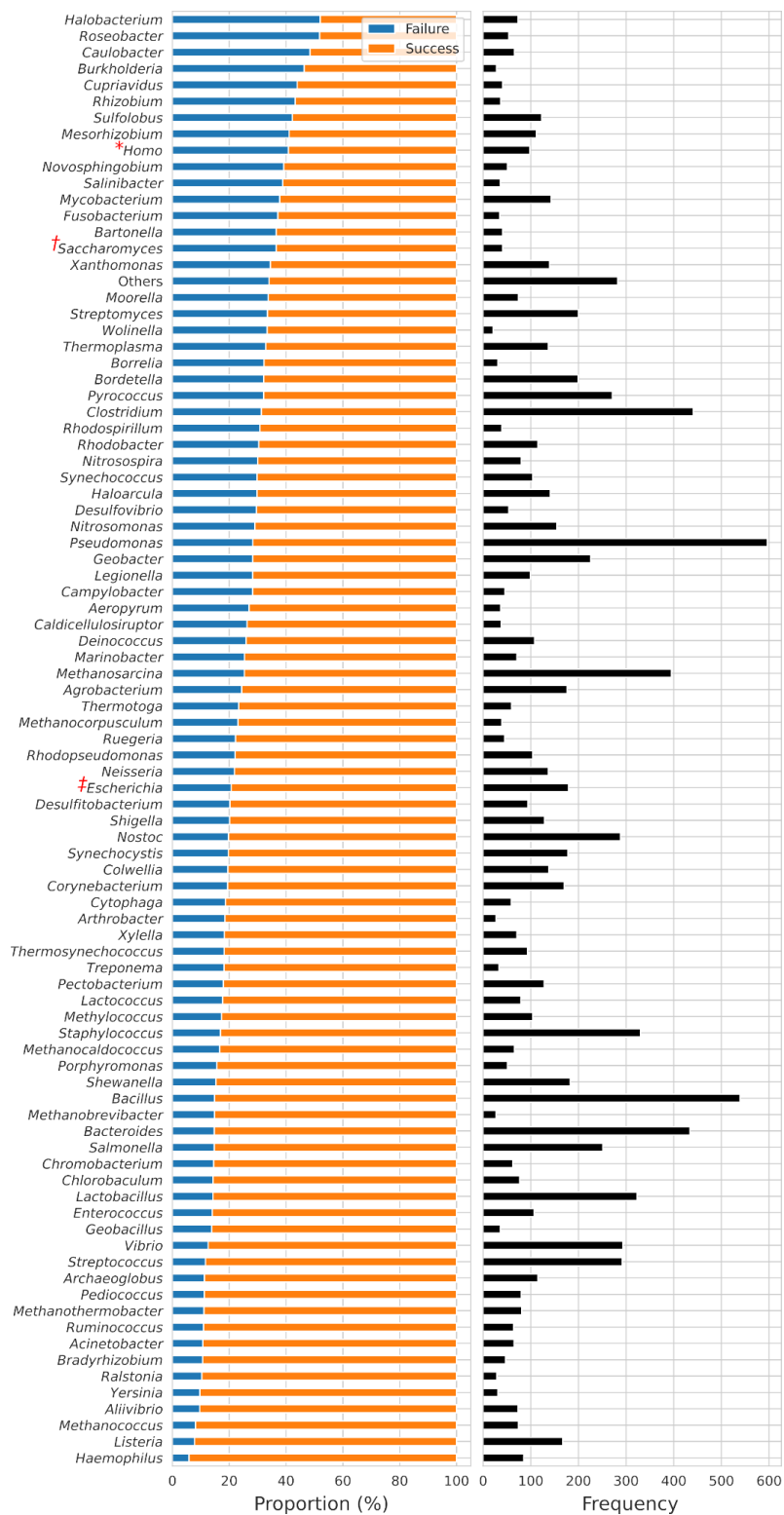

**S2 Fig**

Supplement: S2 Fig — A total of 11,430 PSI:Biology targets from over 189 species were analysed in this study (N = 8,780 and 2,650, ‘success’ and ‘failure’ groups, respectively). Genera with at least 20 target genes are shown and the remaining as ‘Others’. The top three PSI:Biology targets are from four Pseudomonas, five Bacillus and six Clostridium species. Red asterisk, obelisk and diesis indicate Homo sapiens, S. cerevisiae and E. coli, respectively. These target genes were inserted into the pET21_NESG expression vector, in which the promoter and fusion tag are T7lac and C-terminal His tag, respectively. (PDF) [file pcbi.1009461.s002.pdf]

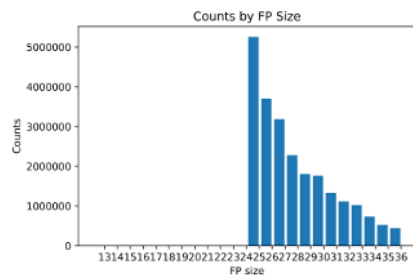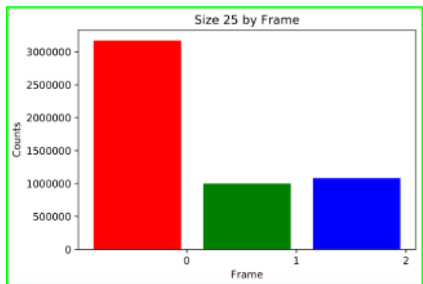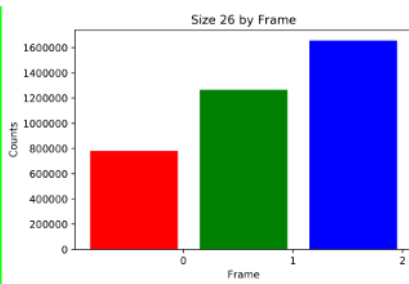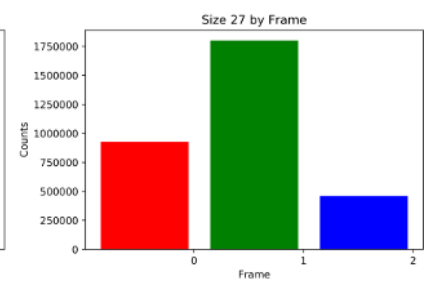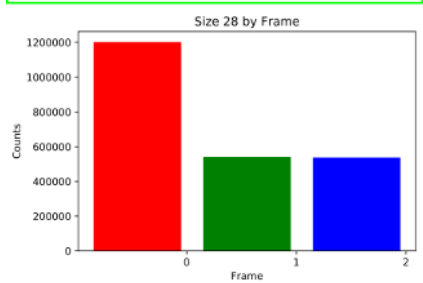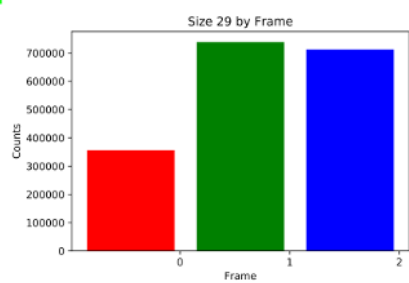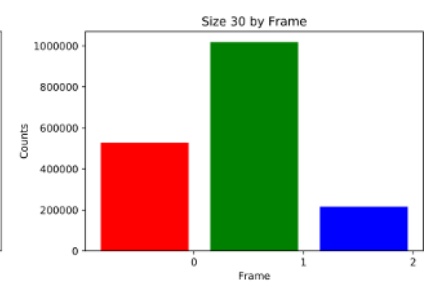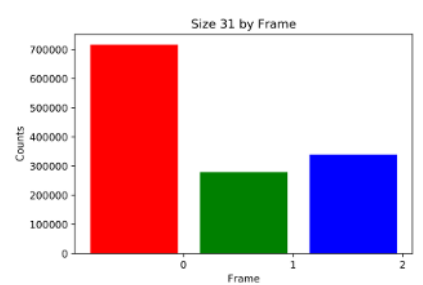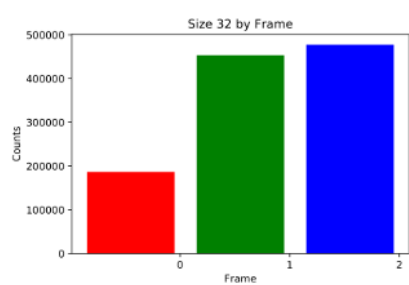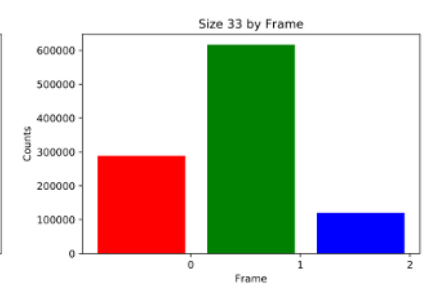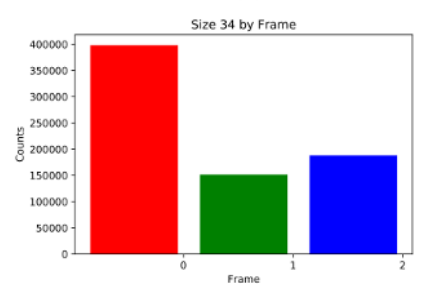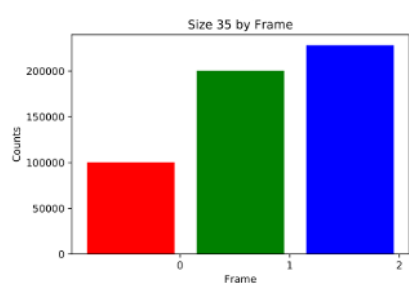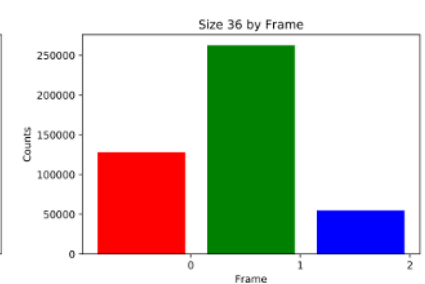

S3 Fig

Supplement: S3 Fig — These 25-nt footprints (green unfilled rectangle) were used to train a neural network model [52] in order to predict the translation elongation rates of the PSI:Biology targets. Ribosome profiling data (SRR7759806 and SRR7759807 [93]) were first aligned to S. cerevisiae transcriptome. SAM alignment files were merged, and ribosome footprints which were mapped to each frame were enumerated. See https://github.com/Gardner-BinfLab/TIsigner_paper_2019. FP, footprints. (PDF) [file pcbi.1009461.s003.pdf]

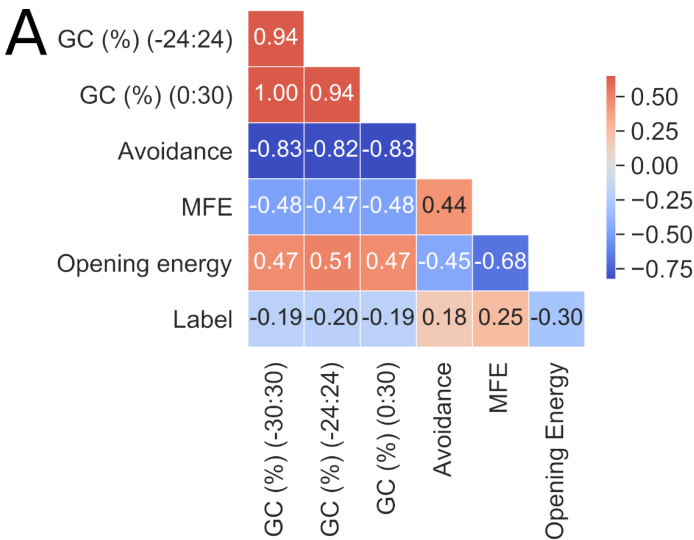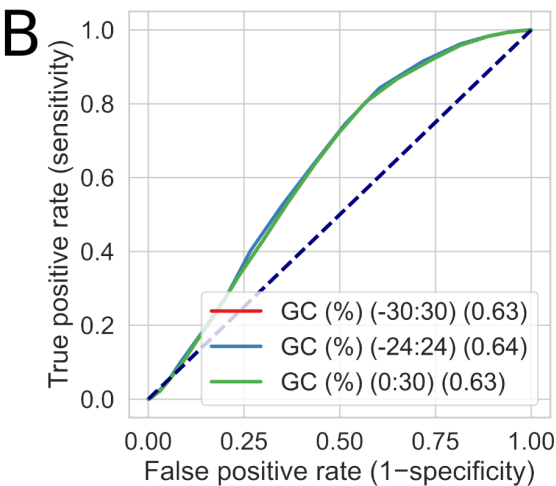

**S4 Fig**

Supplement: S4 Fig — A: The G+C contents in the regions −24:24 and −30:30 weakly correlate with opening energy and MFE, respectively. Green unfilled squares indicate Spearman’s correlations (Rs) between the local G+C contents and the corresponding local features. B: The local G+C contents show a similar prediction accuracy (AUC scores shown in parentheses). AUC, Area Under the receiver operating characteristic Curve; MFE, Minimum Free Energy. (PDF) [file pcbi.1009461.s004.pdf]

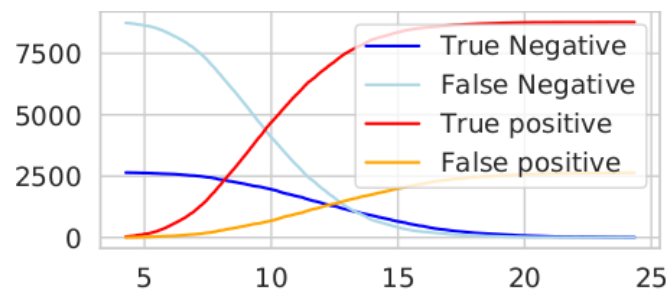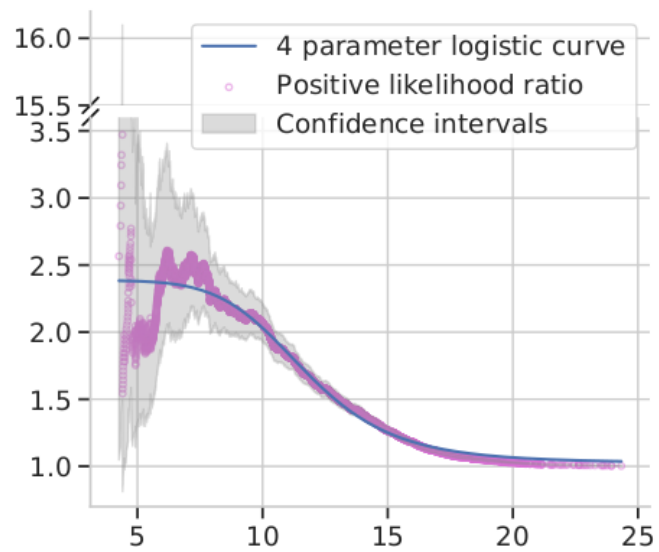

**S5 Fig**

Supplement: S5 Fig — Cumulative frequency distributions of the true positive and false positive (less than type), and true negative and false negative (more than type) derived from the ROC analysis in Fig 4C (left panel, opening energy −24:24). These values were used to estimate positive likelihood ratios with 95% confidence intervals using 10,000 bootstrap replicates. The estimated ratios and/or confidence intervals are inaccurate at low numbers of true positives or true negatives. Therefore, a four-parameter logistic curve was fitted to the positive likelihood ratios. Fitted values are useful to estimate the posterior probability of protein expression. (PDF) [file pcbi.1009461.s005.pdf]

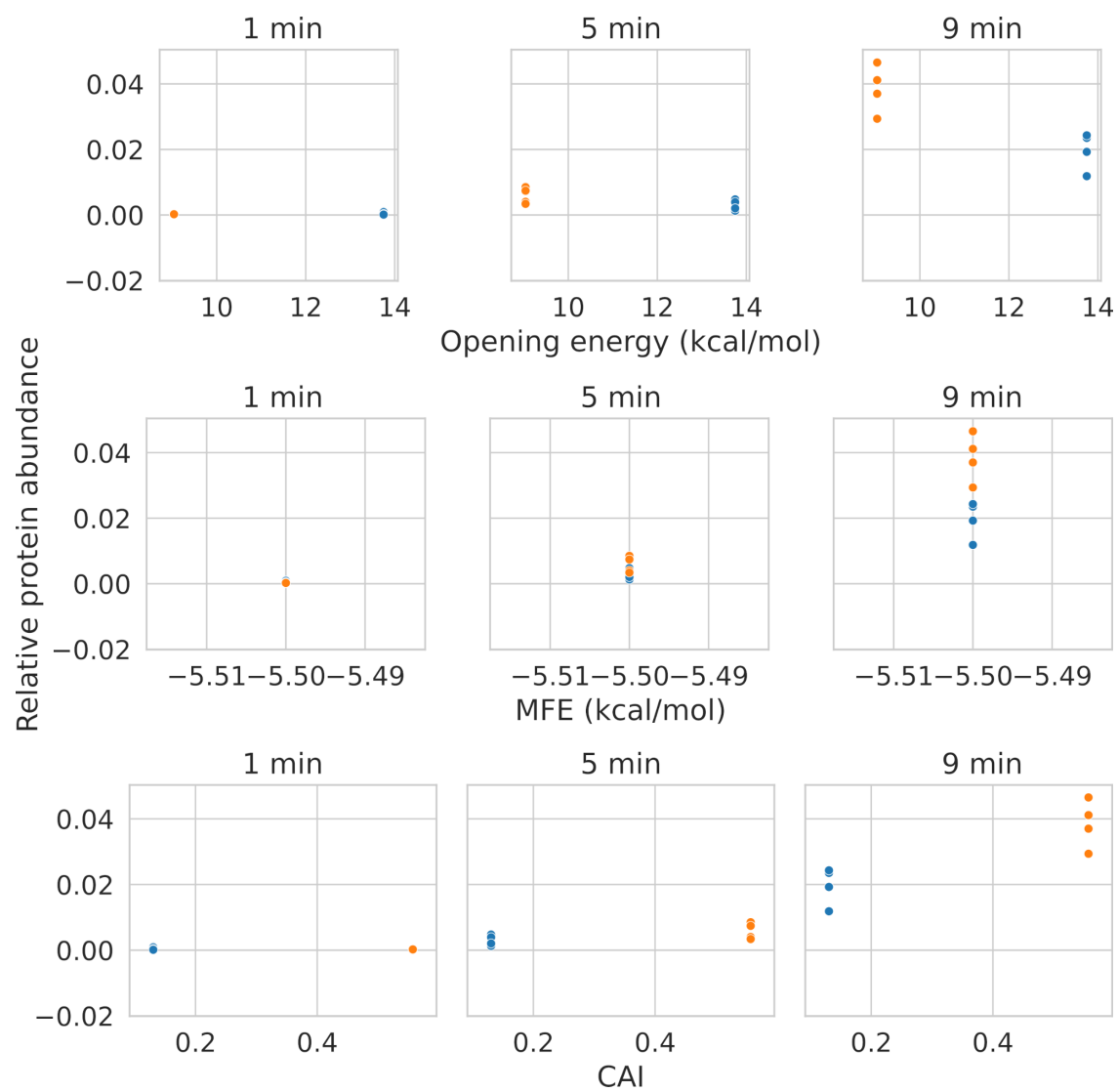

**S6 Fig**

Supplement: S6 Fig — The levels of major capsid protein expressed by the wild-type (orange) and mutant (blue) strains of bacteriophage T7 [53]. The mutant major capsid gene was codon-deoptimised such that the first and the last 14 codons remained unchanged. Proteomic analyses were carried out at 1, 5 and 9 min post-infection in four biological replicates. Opening energy −24:24, MFE −30:30, and CAI of the wild-type and mutant sequences were compared. The approximated ‘Expression Scores’ of the wild-type and mutant sequences are 89 and 38, respectively (opening energies of 9.05 kcal/mol and 13.76 kcal/mol, respectively). MFE, Minimum Free Energy; CAI, Codon Adaptation Index. (PDF) [file pcbi.1009461.s006.pdf]

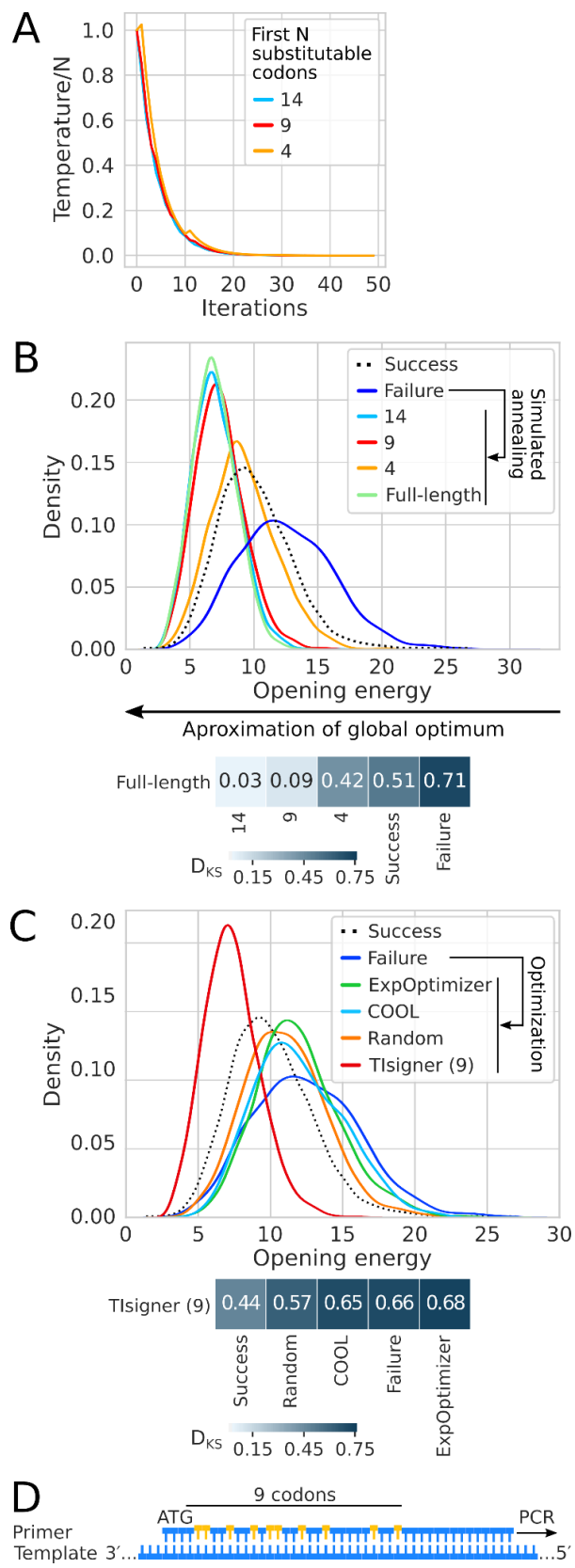

**S7 Fig**

Supplement: S7 Fig — A: Schedules in simulated annealing. The ratio of temperature to the number of the first N substitutable codons decreases exponentially with increasing number of iterations. B: Accessibility of translation initiation sites increases with increasing number of the first N replaceable codons. The PSI:Biology targets that failed to be expressed were optimised using simulated annealing (N = 2,650). The Kolmogorov-Smirnov distance between the distributions of ‘9’ and ‘full-length’ was significantly different but sufficiently close (DKS = 0.09, P < 10−7), indicating that optimisation of the first nine codons can achieve nearly optimum accessibility. For comparison, the distribution of the PSI:Biology targets that were successfully expressed are shown (N = 8,780). See also S1 File. C: Accessibility of translation initiation sites can be increased indirectly using the existing gene optimisation tools and random synonymous codon substitution. ‘TIsigner (9)’ refers to the default settings of our tool, which allows synonymous substitutions up to the first nine codons (as above). D: Accessibility of translation initiation sites can be optimised using PCR. The forward primer should be designed according to TIsiger optimised sequences. For example, using a nested PCR approach, the optimised sequence can be produced using the forward primer designed with appropriate mismatches (gold bulges) to amplify the amplicon from the initial PCR reaction. (PDF) [file pcbi.1009461.s007.pdf]

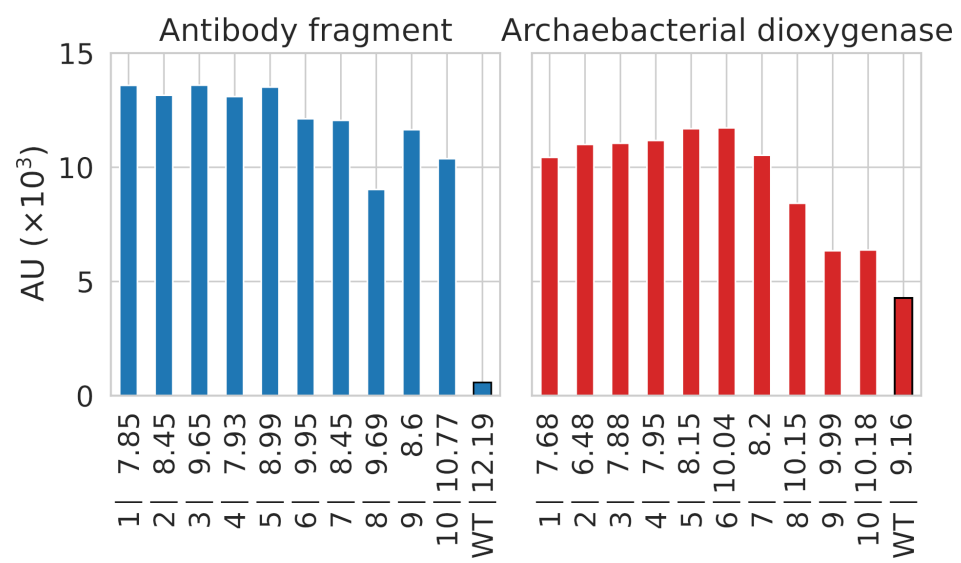

**S9 Fig**

Supplement: S9 Fig — A RTS E. coli cell-free expression system was previously used to express these recombinant proteins [30]. The expression levels are shown in arbitrary units (AU) based on the densitometric analysis of previously published Western blots (S3 File). WT, wild-type. (PDF) [file pcbi.1009461.s009.pdf]

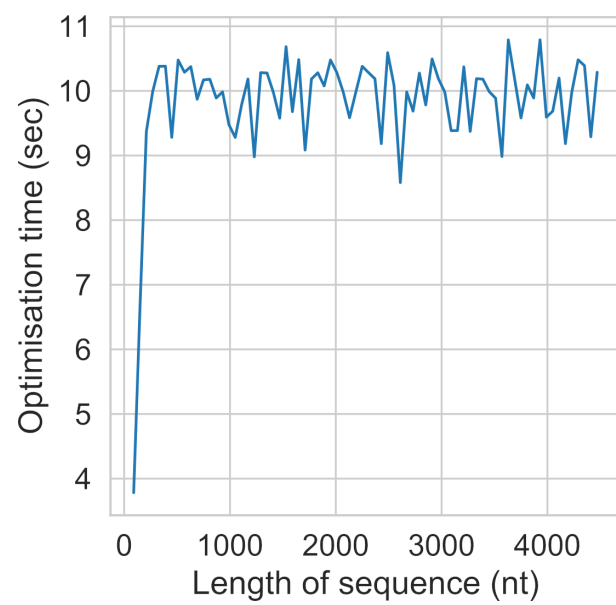

**S10 Fig**

Supplement: S10 Fig — (PDF) [file pcbi.1009461.s010.pdf]
