# Supplementary material for: Analysis of 11,430 recombinant protein production experiments reveals that protein yield is tunable by synonymous codon changes of translation initiation sites
Source: PLoS Comput Biol. 2021 Oct 5;17(10):e1009461. doi: 10.1371/journal.pcbi.1009461 (PMC8519471; doi:10.1371/journal.pcbi.1009461)
Supplement: S8 Fig — A: SDS-PAGE gel shows the protein bands of Renilla luciferase (RLuc) in the soluble and insoluble fractions of BL21Star(DE3) lysates. The expression of RLuc can be improved, despite its poor solubility in E. coli. Selected bacterial clones were grown at 25°C, 200 RPM. The solubilities of wildtype (WT) RLuc and designed variants were compared after 4-hour IPTG induction. The blue and red arrows (about 36kDa) indicate that RLuc was poorly soluble. No RLuc protein bands were detected from the uninduced cultures and IPTG-induced negative control (empty vector control that lacks Rluc gene and T7lac promoter). B: The luciferase activities of commercially designed RLuc reporter genes (full-length sequence optimisation) and a TIsigner optimised sequence (9.9 kcal/mol) are significantly higher than the wild-type luciferase (Mann-Whitney U tests, P = 9.1 × 10^−3^). Opening energies are shown next to labels. IPTG, isopropyl-β-D thiogalactopyranoside. (PDF) [file pcbi.1009461.s008.pdf]

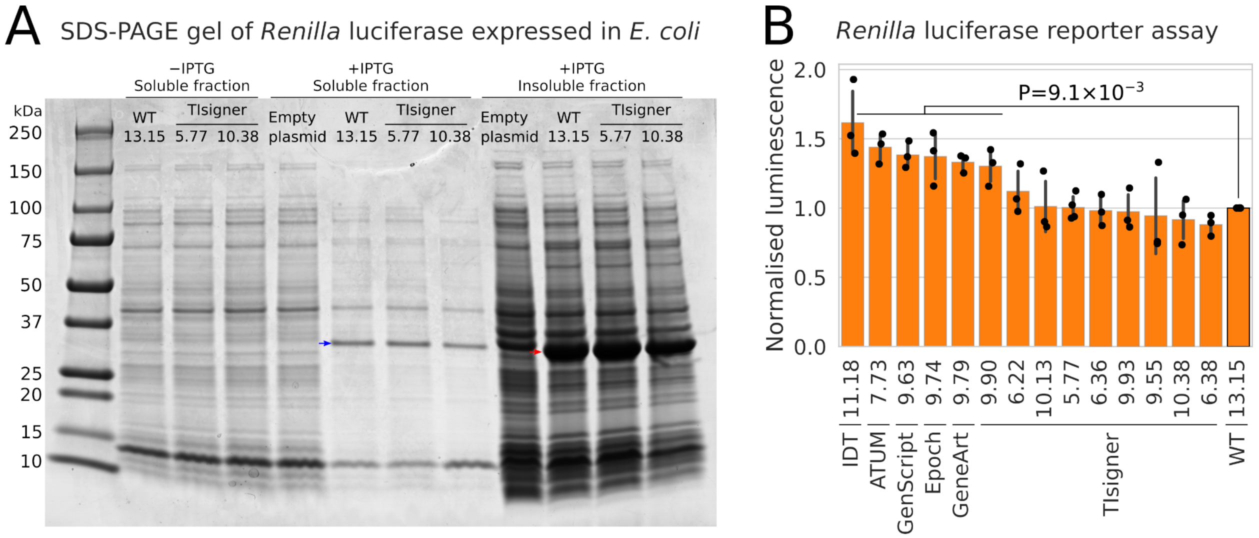
A
SDS-PAGE gel of Renilla luciferase expressed in E. coli
−IPTG
Soluble fraction
+IPTG
Soluble fraction
+IPTG
Insoluble fraction
kDa
WT
13.15
TIsigner
5.77
10.38
Empty
plasmid
WT
13.15
TIsigner
5.77
10.38
Empty
plasmid
WT
13.15
TIsigner
5.77
10.38
250
150
100
75
50
37
25
20
15
10
B
Renilla luciferase reporter assay
Normalised luminescence
2.0
1.5
1.0
0.5
0.0
P=9.1×10−3
IDT
11.18
ATUM
7.73
GenScript
9.63
Epoch
9.74
GeneArt
9.79
TIsigner
9.90
6.22
10.13
5.77
6.36
9.93
9.55
10.38
6.38
WT
13.15

S8 Fig
