# Supplementary material for: Analysis of 11,430 recombinant protein production experiments reveals that protein yield is tunable by synonymous codon changes of translation initiation sites
Source: PLoS Comput Biol. 2021 Oct 5;17(10):e1009461. doi: 10.1371/journal.pcbi.1009461 (PMC8519471; doi:10.1371/journal.pcbi.1009461)
Supplement: S2 File — Figs A-E, and Tables A-C. (PDF) [file pcbi.1009461.s012.pdf]

Additional notes

For a transcript of length n, previous studies have defined 'accessibility' in terms of:

1. Opening energy or base-unpairing probability (as implemented in RNAplfold [1], used in this paper):

   $$\delta G_{openen} = -RTlog(p^{u})$$

   where p^{u} is the probability that the base pairs between $i \ldots j$ is unpaired and is given by:

   $$p^{u} = \frac{Zunpaired(i \ldots j)}{Z(1, n)}$$

   $$= \frac{1}{Z(1, n)}(Z(1, i-1)Z(j+1, n) + \sum_{a \le i} Z^{H}(a, j) + \sum_{a \le i} Z^{I}(a, j) + \sum_{a \le i} Z^{M5}(a, j) + \sum_{a \le i} Z^{M3}(a, j) + \sum_{a \le i} Z^{MB}(a, j)$$

   where, Z^{H}, Z^{I}, Z^{M5}, Z^{M3}, Z^{MB} are the partition function contributions from hairpin, interior loop, multiloop contributions at $5'$ and $3'$ end and unpaired intervals between the multiloop and the closing base pair. A similar base-unpairing approach is also implemented in Raccess [2,3], which further incorporates the stochastic context-free grammar and log-linear models of CONTRAfold [4].

2. Unfolding energy [5]:
   Unfolding energy for the first L bases is defined as:

   $$\Delta G_{unfold}(L) = \Delta G_{1,n} - \Delta G_{L,n},$$

   where, $\Delta G_{1,n}$ is the folding free energy of the entire transcript and $\Delta G_{L,n}$ is the free energy contributions from base-pairings which does not involve the first L bases. These ΔGs are calculated using NUPACK [6].

3. Secondary structure [7,8]:
   Transcripts with stable secondary structures, i.e., low minimum free energy (MFE), are considered less accessible.

4. Base pair probability [9]:
   Transcripts with higher base pairing probability are less accessible. The base pairing probability is calculated using McCaskill's algorithm, rather than unpairing probability.

5. Structure alignment [10]:
   Five sub-sequences of window size (1400 nt) W_{size} with the lowest MFE is aligned. The window is then shifted by 500 nt. The structures of overlapping windows are then further aligned. Repeating this procedure, the accessible motifs were selected only if it is conserved across the windows among several MFE structures.

Accessibility score SC_{acc} is then defined as:

$$SC_{acc} = [S_i \times P_i \times (W_{s1,i}) / 100]/(W_{seq,i} + W_{tot})$$

where S_i is the average length of predicted target motifs, $W_{s1,i}$ is the proportion of analysed windows with the lowest MFE structure $s1$with the motif, $W_{seq,i}$is the number of windows containing the motif and folds into one or more $s1$structures and W_{tot} is the total number of analysed windows.

Additional methods

Cloning of TIsigner variants of GFP and Luciferase

The cloning of TIsigner sequence variants for *Escherichia coli* expression was performed using the MIDAS Golden Gate cloning system [11]. As with other Golden Gate assembly (GGA) systems, MIDAS is a modular, hierarchical DNA assembly system that uses the Type IIS restriction enzymes AarI, BsaI and BsmBI to assemble genes, transcription units and other devices from basic parts, and subsequently enables multiple devices to be assembled together on a single plasmid.

As per MIDAS, basic parts such as promoters, coding sequences and terminators were amplified by PCR or ordered as synthetic polynucleotide sequences from gene synthesis companies. The basic parts are listed in Table A. Protocols for the GGA reactions are as described in van Dolleweerd et al., 2018 [11].

N-terminal region of GFP (GFPN)

Overlapping oligonucleotide primer pairs corresponding to the first ten codons of each of the *gfp* sequence variants produced by the TIsigner algorithm were ordered from Integrated DNA Technologies (IDT) (see Table B). Each overlapping pair of primers was annealed together and used as a template for amplification by Q5 polymerase (New England Biolabs) to create a double-stranded DNA product spanning the N-terminal region of GFP (GFPN; codons 1 to 10; see Fig A), and with MIDAS [CCAT] prefix and [GTTG] suffix nucleotides.

C-terminal region of GFP (GFPC)

The C-terminal region of GFP (designated GFPC), spanning codons 11 to 238 of the native *gfp* of *Aequoria victoria*, was synthesized (GeneArt) with flanking BsmBI recognition sites, and with the [GTTG] MIDAS prefix (compatible with the [GTTG] suffix on the GFPN part) and [GCTT] suffix nucleotides.

N-terminal region of luciferase (RLucN)

Overlapping oligonucleotide primer pairs corresponding to the first ten codons of each of the luciferase sequence variants generated by the TIsigner algorithm were ordered from Integrated DNA Technologies (IDT) (see Table C). Each overlapping pair of primers was annealed together and used as a template for amplification by Q5 polymerase (New England Biolabs) to create a double-stranded DNA product spanning the N-terminal region of luciferase (RLucN; codons 1 to 10), and with a MIDAS [CCAT] prefix and an [AGGA] suffix.

C-terminal region of luciferase (RLucC)
The C-terminal region of luciferase (designated RLucC), spanning codons 11 to 311 of the native luciferase of *Renilla reniformis*, was amplified from the full-length, native luciferase sequence (synthesized by GeneArt) using primers that add flanking BsmBI recognition sites, and a MIDAS [AGGA] prefix (compatible with the [AGGA] suffix on the RLucN part) and a [GCTT] suffix.

<u>MIDAS Level-1 cloning of parts</u>
PCR products, purified using commercially available column-based protocols (Macherey-Nagel), or parts produced by gene synthesis were cloned into the MIDAS pML1 vector by BsmBI-mediated Golden Gate assembly (BsmBI-GGA). As per the MIDAS design, BsmBI-GGA into the pML1 vector results in elimination of the BsmBI recognition sites and each part becomes flanked by BsaI recognition sites that cleave at the MIDAS prefix and suffix nucleotides.

In the case of the *Aequoria victoria gfp* TIsigner variants, each GFPN part cloned into the pML1 vector becomes flanked by BsaI recognition sites that are cleaved at the [CCAT] prefix and [GTTG] suffix (Fig B). Cloning of the GFPC part into the pML1 vector results in a GFPC module flanked by BsaI recognition sites that are cleaved at the [GTTG] prefix and at the [GCTT] suffix (Fig C).

In the case of the *Renilla reniformis* luciferase TIsigner variants, each RLucN part cloned into the pML1 vector becomes flanked by BsaI recognition sites that are cleaved at the [CCAT] prefix and [AGGA] suffix, while the C-terminal fragment, RLucC, becomes flanked by BsaI recognition sites that generate an [AGGA] prefix and a [GCTT] suffix upon cleavage.

All parts cloned into the pML1 vector were verified by sequencing.

<u>MIDAS Level-2 assembly of devices</u>
Devices were assembled from the cloned Level-1 modules described above, using BsaI-GGA, into the appropriate pML2 vector. As per the MIDAS design, multiple parts can be assembled together, with the position of each part in the assembled device dictated by the compatibility of the prefix and suffix nucleotides flanking each module:

- A *lacI* device was assembled in pML2(+)WR from the single *lacI* genetic element module.
- An *mScarlet-I* device was assembled in pML2(+)BR from *nptII* promoter, *mScarlet-I* CDS and lambda t0 transcription terminator modules.
- Full-length *gfp* devices for each TIsigner variant were assembled in pML2(+)WF from the following modules: *T7lac* promoter, GFPN, GFPC and T7 Tφ transcription terminator. Since the prefix of the GFPC module, [GTTG] (see Fig C), is identical to the suffix of each GFPN module (see Fig B) this allows the two modules to be genetically fused so that, together with the *T7lac* promoter and T7 Tφ transcription terminator modules, full-length *gfp* devices are assembled for each variant. The *mScarlet-I* and *gfp* devices were assembled in pML2 vectors of opposite orientation (using the “Reverse” vector pML2(+)BR for *mScarlet-I*, and the “Forward” vector pML2(+)WF for each *gfp* device), so

that they will be divergently transcribed once assembled into the expression vector (Level-3, see below).

- In a similar fashion, full-length luciferase devices for each TIsigner variant were assembled in pML2(+)BF from *T7lac* promoter, RLucN, RLucC and T7 Tφ transcription terminator modules.

All cloned devices were verified by restriction mapping and sequencing.

MIDAS Level-3 assembly (construction of the expression plasmids)

E. coli gfp expression plasmids were constructed by sequentially loading the *lacI*, *mScarlet-I* and *gfp* devices, using alternating AarI- and BsmBI-GGA reactions, into the MIDAS Level-3 destination plasmid pML3.2, which has the medium copy replication origin from the pET series of vectors in place of the high copy pMB1 replicon of the pML3 destination vector originally described in van Dolleweerd et al, 2018 [11]. A representative map of an *E. coli* expression plasmid containing all three devices is shown in Fig D.
For luciferase expression, the intermediate plasmid containing the *lacI* device (described above) was used for assembly of each of the luciferase devices (i.e., no *mScarlet-I* device was added), and a representative map of an *E. coli* plasmid for luciferase expression is shown in Fig E. The *lacI* and luciferase devices are divergently transcribed from the expression vector.

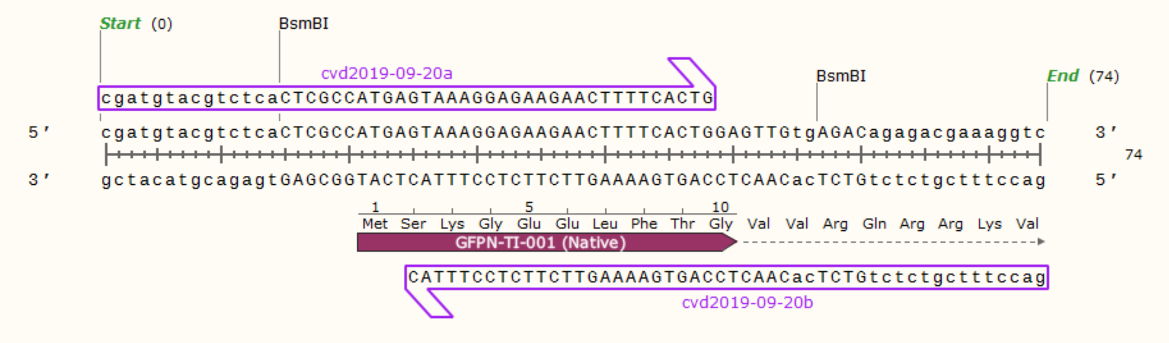

Fig A. Strategy for producing a double stranded DNA corresponding to the first ten codons of each of the TIsigner variants of GFP. The strategy employs a pair of primers that overlap at their 3’ ends that, upon annealing together, can be used as a template for Q5 polymerase to generate a double stranded DNA spanning the N-terminal region of GFP (i.e. GFPN). Shown here is the sequence of variant GFPN-001 generated using the overlapping cvd2019-09-20a forward and cvd2019-09-20b reverse primer pair (see Table B). The resultant double stranded DNA can then be cloned into the MIDAS pML1 vector by digestion with the Type IIS restriction enzyme BsmBI (recognition site <u>CGTCTC</u>(1/5)). The same primer pair strategy was used for producing TIsigner variants of luciferase, albeit with a different [AGGA] suffix. This map was created with SnapGene.

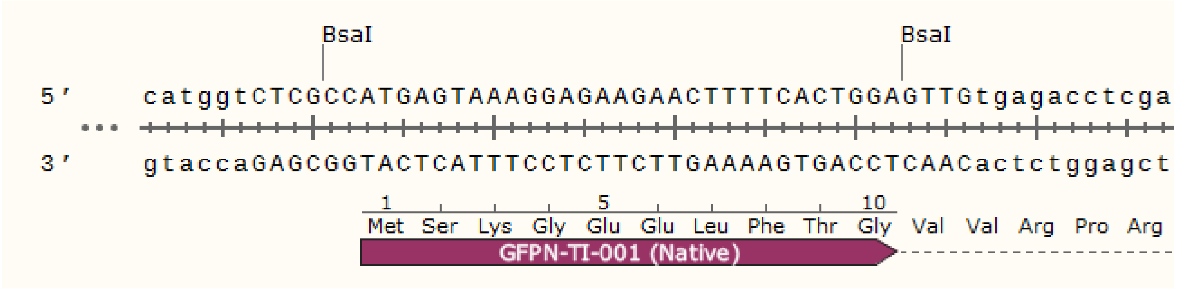

Fig B. Structure of GFPN variants cloned into the pML1 vector. Following BsmBI-mediated cloning into the pML1 vector, each GFPN variant becomes flanked by BsaI recognition sites (GGTCTC(1/5)). BsaI cleaves at the CCAT prefix upstream of the GFPN sequence and at the GTTG suffix (downstream of the GFPN module). For ease of depiction, only the sequences immediately surrounding the cloned GFPN fragment are shown (i.e., not the rest of the pML1 vector). The structure of luciferase RLucN variants is identical, except for having a different [AGGA] suffix sequence.

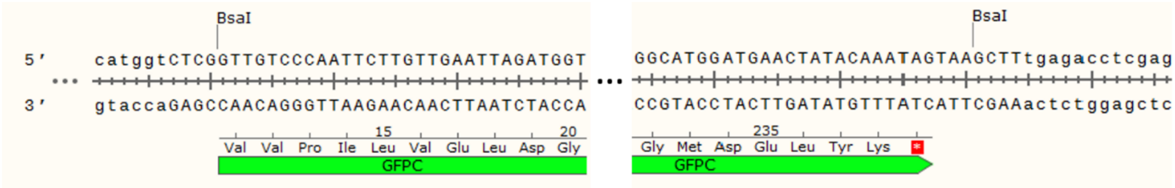

Fig C. Structure of the GFPC fragment cloned into the pML1 vector. Following BsmBI-mediated cloning into the pML1 vector, GFPC becomes flanked by BsaI recognition sites. BsaI cleaves at the [GTTG] prefix (upstream of the GFPC sequence) and at the downstream [GCTT] suffix. For ease of depiction, only sequences around the 5' and 3' ends of the GFPC fragment are shown (left- and right-hand sides, respectively). In the case of luciferase, the prefix sequence is [AGGA].

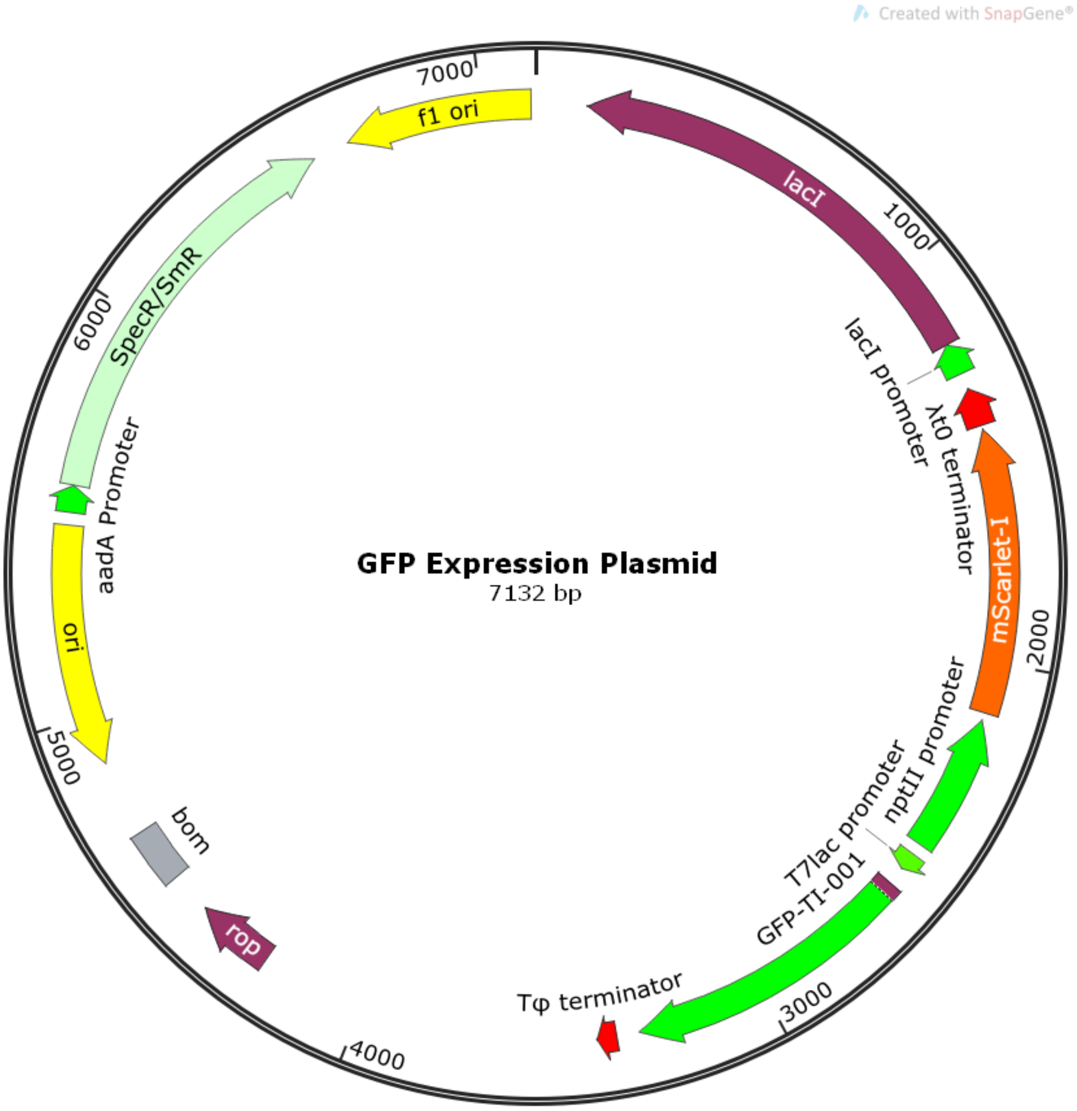

Fig D. Structure of GFP expression plasmids. Map view showing the architecture of MIDAS-assembled plasmids used for expression of GFP TIsigner variants. Expression of each of the gfp variants is controlled by the *T7lac* promoter and oriented such that they are divergently transcribed with respect to the *mScarlet-I* device, which is driven by the *nptII* promoter. The devices for *lacI*, *mScarlet-I* and *gfp* were loaded sequentially into plasmid pML3.2, which has the medium copy replication origin from the pET series of vectors (*ori-bom-rop*) in place of the high copy pMB1 replicon in the pML3 destination vector described in van Dolleweerd et al, 2018 [11], and a selectable marker conferring resistance to spectinomycin.

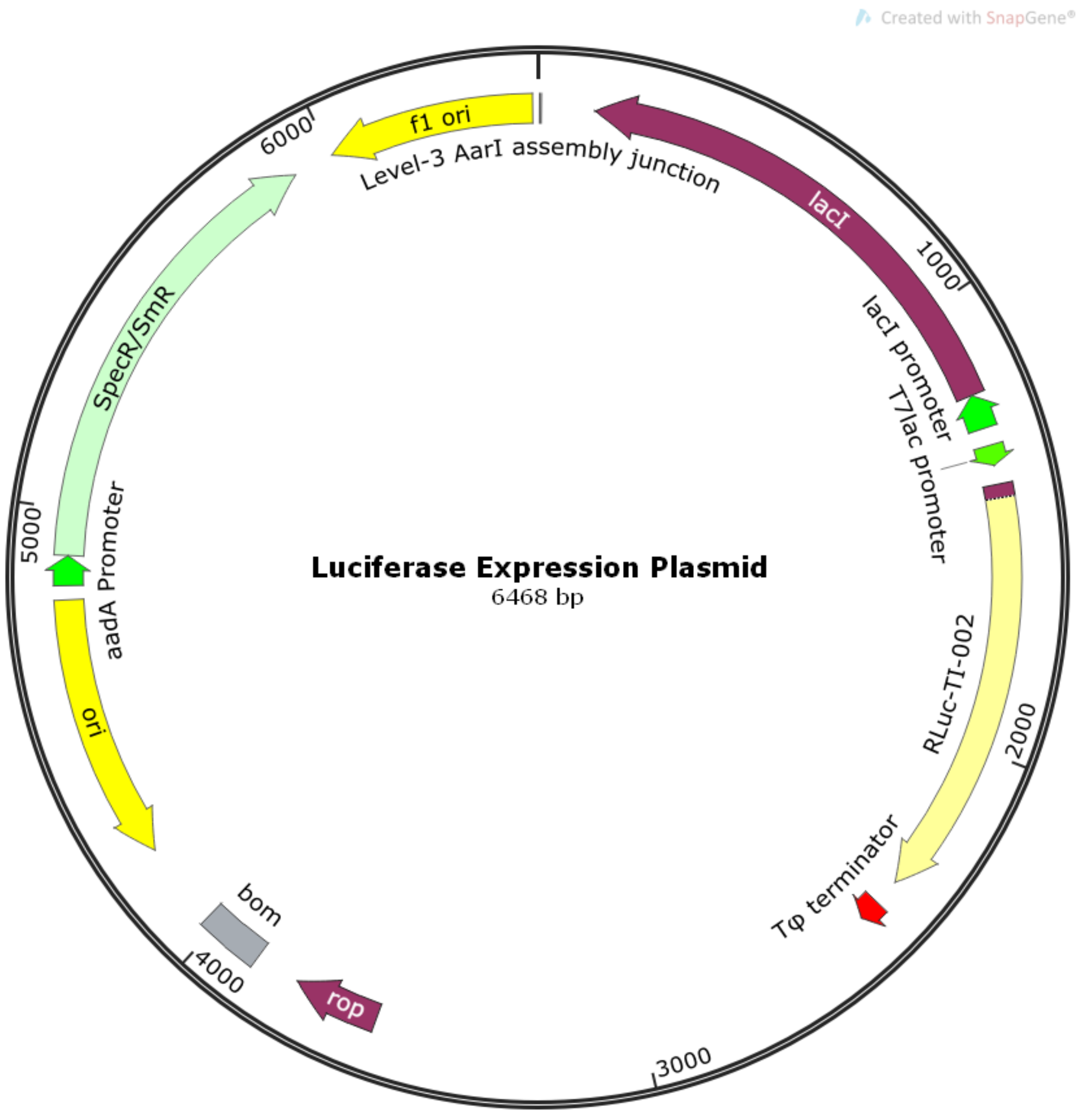

Fig E. Structure of luciferase expression plasmids. Map view showing the architecture of MIDAS-assembled plasmids used for expression of luciferase TIsigner variants. Expression of each of the luciferase variants is controlled by the *T7lac* promoter and oriented such that they are divergently transcribed with respect to the *lacI* device, which is driven by the *lacI* promoter. The devices for *lacI* and luciferase were loaded sequentially into plasmid pML3.2, which has the medium copy replication origin from the pET series of vectors (*ori-bom-rop*) in place of the high copy pMB1 replicon in the pML3 destination vector described in van Dolleweerd et al, 2018 [11], and a selectable marker conferring resistance to spectinomycin.

Table A. MIDAS parts used in this work. The sequence of each part is shown, with BsmBI recognition sites used for cloning into the MIDAS pML1 vector underlined. MIDAS prefix and suffix nucleotides are highlighted by the bold blue and red text, respectively. The *T7lac* promoter and T7 Tφ transcription terminator (both sequences taken from pET-15b) were ordered as double-stranded DNA gBlocks from IDT. The *lacI* genetic element (with *lacI* promoter in green and lacI coding sequence in blue) was from GeneArt. Other parts were amplified by polymerase chain reaction. For the *mScarlet-I* part, the coding sequence is highlighted by the blue shading. Sequences of all parts were confirmed following cloning into the MIDAS pML1 vector.

Part	Sequence
T7lac promoter	cgatgtacgtctcaCTCG**GGAG**CGATCCCGCGAAATTAATACGACTCACTAT<br>AGGGGAATTGTGAGCGGATAACAATTCCCCTCTAGAAATAATTTTGTT<br>TAACTTTAAGAAGGAGATATA**CCAT**tgAGACagagacgaaaggtc
nptII promoter	cgatgtacgtctcaCTCG**GGAG**ctcgcacgctgccgcaagcactcagggcgcaagggctgct<br>aaaggaagcggaacacgtagaaagccagtccgcagaaacggtgctgaccccggatgaatgtca<br>gctactgggctatctggacaagggaaaacgcaagcgcaaagagaaagcaggtagcttgcagtgg<br>gcttacatggcgatagctagactgggcggtcttatggacagcaagcgaaccggaattgccagctgg<br>ggcgccctctggtaaggttgggaagccctgcaaagtaaactggatggctttcttgccgccaaggatct<br>gatggcgcaggggatcaagatctgatcaagagacaggatactagtggaggaagaaaa**AATG**t<br>gAGACagagacgaaaggtc
lacI genetic element	cgatgtacgtctcaCTCG**GGAG**CGTCGAGATCCCGGACACCATCGAATGG<br>CGCAAAACCTTTCGCGGTATGGCATGATAGCGCCCGGAAGAGAGTC<br>AATTCAGGGTGGTGAATGTGAAACCAGTAACGTTATACGATGTCGCA<br>GAGTATGCCGGTGTCTCTTATCAGACCGTTTCCCGCGTGGTGAACC<br>AGGCCAGCCACGTTTCTGCGAAAACGCGGGAAAAAGTGGAAGCGG<br>CGATGGCGGAGCTGAATTACATTCCCAACCGCGTGGCACAACAACT<br>GGCGGGCAAACAGTCGTTGCTGATTGGCGTTGCCACCTCCAGTCTG<br>GCCCTGCACGCGCCGTCGCAAATTGTCGCGGCGATTAAATCTCGCG<br>CCGATCAACTGGGTGCCAGCGTGGTGGTGTCGATGGTAGAACGAAG<br>CGGCGTCGAAGCCTGTAAAGCGGCGGTGCACAATCTTCTCGCGCAA<br>CGCGTCAGTGGGCTGATCATTAACTATCCGCTGGATGACCAGGATGC<br>CATTGCTGTGGAAGCTGCCTGCACTAATGTTCCGGCGTTATTTCTTG<br>ATGTCTCTGACCAGACACCCATCAACAGTATTATTTTCTCCCATGAAG<br>ACGGTACGCGACTGGGCGTGGAGCATCTGGTCGCATTGGGTCACC<br>AGCAAATCGCGCTGTTAGCGGGCCCATTAAGTTCTGTCTCGGCGCG<br>TCTGCGTCTGGCTGGCTGGCATAAATATCTCACTCGCAATCAAATTCA<br>GCCGATAGCGGAACGGGAAGGCGACTGGAGTGCCATGTCCGGTTT<br>TCAACAAACCATGCAAATGCTGAATGAGGGCATCGTTCCCACTGCGA<br>TGCTGGTTGCCAACGATCAGATGGCGCTGGGCGCAATGCGCGCCAT<br>TACCGAGTCCGGGCTGCGCGTTGGTGCGGATATCTCGGTAGTGGGA<br>TACGACGATACCGAAGACAGCTCATGTTATATCCCGCCGTTAACCAC<br>CATCAAACAGGATTTTCGCCTGCTGGGGCAAACCAGCGTGGACCGC<br>TTGCTGCAACTCTCTCAGGGCCAGGCGGTGAAGGGCAATCAGCTGT<br>TGCCCGTGTCACTGGTGAAAAGAAAAACCACCCTGGCGCCCAATAC<br>GCAAACCGCCTCTCCCCGCGCGTTGGCCGATTCATTAATGCAGCTG<br>GCACGACAGGTTTCCCGACTGGAAAGCGGGCAGTGAGCGCAACGC<br>AATTAATGTAAGTTAGCTCACTCATTAGGCACCGGGATCTCGACCGAT

	GCCCTTGAGAGCCTTCAACCCAGTCAGCTCCTTCCGGTGGGCGCG<br>GGGCATGACTA**CGCT**tgAGACagagacgaaaggtc
mScarlet-I coding sequence (CDS)	cgatgtacgtctcaCTCG**AATG**gtgagcaagggcgaggcagtgatcaaggagttcatgcggtt<br>caaggtgcacatggagggctccatgaacggccacgagttcgagatcgagggcgagggcgaggg<br>ccgcccctacgagggcacccagaccgccaagctgaaggtgaccaagggtggccccctgcccttct<br>cctgggacatcctgtcccctcagttcatgtacggctccagggccttcatcaagcaccccgccgacatc<br>cccgactactataagcagtccttccccgagggcttcaagtgggagcgcgtgatgaacttcgaggac<br>ggcggcgccgtgaccgtgacccaggacacctccctggaggacggcaccctgatctacaaggtga<br>agctccgcggcaccaacttccctcctgacggccccgtaatgcagaagaagacaatgggctgggaa<br>gcgtccaccgagcggttgtaccccgaggacggcgtgctgaagggcgacattaagatggccctgcg<br>cctgaaggacggcggccgctacctggcggacttcaagaccacctacaaggccaagaagcccgtg<br>cagatgcccggcgcctacaacgtcgaccgcaagttggacatcacctcccacaacgaggactaca<br>ccgtggtggaacagtacgaacgctccgagggccgccactccaccggcggcatggacgagctgta<br>caagtaa**GCTT**tgAGACagagacgaaaggtc
Phage T7 Tφ transcription terminator	cgatgtacgtctcaCTCG**GCTT**CAAAGCCCGAAAGGAAGCTGAGTTGGCT<br>GCTGCCACCGCTGAGCAATAACTAGCATAACCCCTTGGGGCCTCTAA<br>ACGGGTCTTGAGGGGTTTTTTGCTGAAAGGAGGAACTATATCCGGAT<br>**CGCT**tgAGACagagacgaaaggtc
Lambda t0 transcription terminator	cgatgtacgtctcaCTCG**GCTT**ggactcctgttgatagatccagtaatgacctcagaactccatct<br>ggatttgttcagaacgctcggttgccgccgggcgtttttattggtgagaatccaagctagcttgg**CGC**<br>**T**tgAGACagagacgaaaggtc

Table B. Oligonucleotide primer pairs for constructing TIsigner variants of *gfp*. The sequences of each forward and reverse primer pair used for constructing each of the *gfp* TIsigner variants is shown. The start codon in each of the forward primers is shaded yellow. BsmBI recognition sites (used for Golden Gate assembly into the MIDAS pML1 vector) are underlined. The MIDAS prefix [CCAT] and suffix [GTTG] (reverse-complement = CAAC) for the GFPN modules are highlighted in bold blue and red, respectively.

GFPN TIsigner ID	Oligonucleotide Primer Pair	Primer Sequences (5' to 3')
GFPN-001	cvd2019-09-20a	cgatgtacgtctcaCTCG**CCAT**GAGTAAAGGAGAAGAACTTTTCACTG
	cvd2019-09-20b	gacctttcgtctctGTCTca**CAAC**TCCAGTGAAAAGTTCTTCTCCTTTAC
GFPN-002	cvd2019-09-21a	cgatgtacgtctcaCTCG**CCAT**GTCGAAGGGTGAAGAACTCTTCAC
	cvd2019-09-21b	gacctttcgtctctGTCTca**CAAC**ACCAGTGAAGAGTTCTTCACCCTTC
GFPN-003	cvd2019-09-21c	cgatgtacgtctcaCTCG**CCAT**GAGTAAAGGGGAGGAACTCTTTAC
	cvd2019-09-21d	gacctttcgtctctGTCTca**CAAC**CCCGGTAAAGAGTTCCTCCCCTTTAC
GFPN-004	cvd2019-09-21e	cgatgtacgtctcaCTCG**CCAT**GTCGAAGGGCGAAGAACTCTTC
	cvd2019-09-21f	gacctttcgtctctGTCTca**CAAC**ACCAGTGAAGAGTTCTTCGCCCTTC
GFPN-005	cvd2019-09-21g	cgatgtacgtctcaCTCG**CCAT**GTCTAAGGGTGAGGAGCTCTTC
	cvd2019-09-21h	gacctttcgtctctGTCTca**CAAC**TCCCGTGAAGAGCTCCTCACCCTTAG
GFPN-006	cvd2019-09-21i	cgatgtacgtctcaCTCG**CCAT**GTCGAAAGGGGAAGAACTGTTCAC
	cvd2019-09-21j	gacctttcgtctctGTCTca**CAAC**GCCGGTGAACAGTTCTTCCCCTTTC
GFPN-007	cvd2019-09-21k	cgatgtacgtctcaCTCG**CCAT**GTCTAAAGGAGAAGAGCTTTTCAC
	cvd2019-09-21l	gacctttcgtctctGTCTca**CAAC**CCCAGTGAAAAGCTCTTCTCCTTTAG
GFPN-008	cvd2019-09-21m	cgatgtacgtctcaCTCG**CCAT**GAGTAAGGGTGAGGAATTATTCACG
	cvd2019-09-21n	gacctttcgtctctGTCTca**CAAC**GCCCGTGAATAATTCCTCACCCTTAC
GFPN-009	cvd2019-09-22a	cgatgtacgtctcaCTCG**CCAT**GAGTAAAGGGGAAGAACTGTTCAC
	cvd2019-09-22b	gacctttcgtctctGTCTca**CAAC**GCCAGTGAACAGTTCTTCCCCTTTAC
GFPN-010	cvd2019-09-22c	cgatgtacgtctcaCTCG**CCAT**GTCTAAGGGTGAGGAGCTCTTC
	cvd2019-09-22d	gacctttcgtctctGTCTca**CAAC**TCCTGTGAAGAGCTCCTCACCCTTAG
GFPN-011	cvd2019-09-22e	cgatgtacgtctcaCTCG**CCAT**GAGTAAAGGAGAAGAGTTATTTACTGG
	cvd2019-09-22f	gacctttcgtctctGTCTca**CAAC**TCCAGTAAATAACTCTTCTCCTTTAC
GFPN-012	cvd2019-09-22g	cgatgtacgtctcaCTCG**CCAT**GAGTAAGGGAGAAGAGCTGTTC

	cvd2019-09-22h	gacctttcgtctctGTCTcaCAACTCCAGTGAACAGCTCTTCTCCCTTAC
GFPN-013	cvd2019-09-22i	cgatgtacgtctcaCTCGCCATGTCGAAAGGAGAAGAATTGTTCAC
	cvd2019-09-22j	gacctttcgtctctGTCTcaCAACGCCCGTGAACAATTCTTCTCCTTTCG
GFPN-014	cvd2019-09-22k	cgatgtacgtctcaCTCGCCATGAGCAAAGGAGAAGAATTATTTACTGG
	cvd2019-09-22l	gacctttcgtctctGTCTcaCAACTCCAGTAAATAATTCTTCTCCTTTGC
GFPN-015	cvd2019-09-22 m	cgatgtacgtctcaCTCGCCATGAGCAAAGGAGAAGAATTATTTACGG
	cvd2019-09-22n	gacctttcgtctctGTCTcaCAACTCCCGTAAATAATTCTTCTCCTTTGC
GFPN-016	cvd2019-09-22o	cgatgtacgtctcaCTCGCCATGAGCAAAGGGGAAGAATTATTTACAG
	cvd2019-09-22p	gacctttcgtctctGTCTcaCAACACCTGTAAATAATTCTTCCCCTTTGC
GFPN-017	cvd2020-03-05a	cgatgtacgtctcaCTCGCCATGAGTAAAGGGGAAGAACTCTTTACC
	cvd2020-03-05b	gacctttcgtctctGTCTcaCAACCCCGGTAAAGAGTTCTTCCCCTTTAC
GFPN-018	cvd2020-03-05c	cgatgtacgtctcaCTCGCCATGTCGAAAGGTGAGGAACTATTCACTG
	cvd2020-03-05d	gacctttcgtctctGTCTcaCAACACCAGTGAATAGTTCCTCACCTTTC
GFPN-019	cvd2020-03-05e	cgatgtacgtctcaCTCGCCATGTCGAAGGGTGAAGAACTGTTCACTG
	cvd2020-03-05f	gacctttcgtctctGTCTcaCAACACCAGTGAACAGTTCTTCACCCTTC
GFPN-020	cvd2020-03-05g	cgatgtacgtctcaCTCGCCATGTCGAAGGGTGAAGAACTTTTCACTG
	cvd2020-03-05h	gacctttcgtctctGTCTcaCAACCCCAGTGAAAAGTTCTTCACCCTTC
GFPN-021	cvd2020-03-05i	cgatgtacgtctcaCTCGCCATGTCCAAAGGGGAGGAACTCTTTACG
	cvd2020-03-05j	gacctttcgtctctGTCTcaCAACGCCCGTAAAGAGTTCCTCCCCTTTG
GFPN-022	cvd2020-03-05k	cgatgtacgtctcaCTCGCCATGTCCAAAGGTGAAGAGCTTTTCACC
	cvd2020-03-05l	gacctttcgtctctGTCTcaCAACCCCGGTGAAAAGCTCTTCACCTTTG
GFPN-023	cvd2020-03-05 m	cgatgtacgtctcaCTCGCCATGTCGAAAGGTGAAGAGCTGTTCAC
	cvd2020-03-05n	gacctttcgtctctGTCTcaCAACACCGGTGAACAGCTCTTCACCTTTC
GFPN-024	cvd2020-03-05o	cgatgtacgtctcaCTCGCCATGTCGAAAGGTGAGGAACTGTTCAC
	cvd2020-03-05p	gacctttcgtctctGTCTcaCAACCCCAGTGAACAGTTCCTCACCTTTC
GFPN-025	cvd2020-03-05q	cgatgtacgtctcaCTCGCCATGAGTAAGGGGGAGGAGCTCTTCAC
	cvd2020-03-05r	gacctttcgtctctGTCTcaCAACTCCGGTGAAGAGCTCCTCCCCCTTA C

GFPN-026	cvd2020-03-05s	cgatgtacgtctcaCTCGCCATGAGTAAAGGGGAAGAGCTTTTCAC
	cvd2020-03-05t	gacctttcgtctctGTCTcaCAACCCCGGTGAAAAGCTCTTCCCCTTTAC
GFPN-027	cvd2020-03-06a	cgatgtacgtctcaCTCGCCATGAGTAAAGGAGAAGAACTCTTTACCG
	cvd2020-03-06b	gacctttcgtctctGTCTcaCAACTCCGGTAAAGAGTTCTTCTCCTTTAC
GFPN-028	cvd2020-03-06c	cgatgtacgtctcaCTCGCCATGAGTAAAGGAGAAGAACTCTTCACC
	cvd2020-03-06d	gacctttcgtctctGTCTcaCAACACCGGTGAAGAGTTCTTCTCCTTTAC
GFPN-029	cvd2020-03-06e	cgatgtacgtctcaCTCGCCATGTCAAAGGGGGAAGAACTGTTCAC
	cvd2020-03-06f	gacctttcgtctctGTCTcaCAACGCCTGTGAACAGTTCTTCCCCCTTTG
GFPN-030	cvd2020-03-06g	cgatgtacgtctcaCTCGCCATGTCGAAAGGCGAGGAACTGTTCAC
	cvd2020-03-06h	gacctttcgtctctGTCTcaCAACTCCAGTGAACAGTTCCTCGCCTTTC
GFPN-031	cvd2020-03-06i	cgatgtacgtctcaCTCGCCATGAGCAAGGGTGAAGAGTTATTCACTG
	cvd2020-03-06j	gacctttcgtctctGTCTcaCAACTCCAGTGAATAACTCTTCACCCTTG
GFPN-032	cvd2020-03-06k	cgatgtacgtctcaCTCGCCATGTCTAAAGGTGAAGAACTATTCACAG G
	cvd2020-03-06l	gacctttcgtctctGTCTcaCAACCCCTGTGAATAGTTCTTCACCTTTAG
GFPN-033	cvd2020-03-06 m	cgatgtacgtctcaCTCGCCATGTCTAAAGGTGAGGAGCTCTTCAC
	cvd2020-03-06n	gacctttcgtctctGTCTcaCAACTCCTGTGAAGAGCTCCTCACCTTTAG
GFPN-034	cvd2020-03-06o	cgatgtacgtctcaCTCGCCATGAGTAAGGGAGAGGAACTGTTCAC
	cvd2020-03-06p	gacctttcgtctctGTCTcaCAACCCCTGTGAACAGTTCCTCTCCCTTAC
GFPN-035	cvd2020-03-06q	cgatgtacgtctcaCTCGCCATGTCGAAAGGGGAAGAATTGTTCAC
	cvd2020-03-06r	gacctttcgtctctGTCTcaCAACTCCAGTGAACAATTCTTCCCCTTTCG
GFPN-036	cvd2020-03-06s	cgatgtacgtctcaCTCGCCATGAGTAAGGGGGAGGAGCTGTTC
	cvd2020-03-06t	gacctttcgtctctGTCTcaCAACTCCTGTGAACAGCTCCTCCCCCTTAC
GFPN-037	cvd2020-03-07a	cgatgtacgtctcaCTCGCCATGAGTAAGGGAGAGGAATTGTTCAC
	cvd2020-03-07b	gacctttcgtctctGTCTcaCAACACCCGTGAACAATTCCTCTCCCTTAC
GFPN-038	cvd2020-03-07c	cgatgtacgtctcaCTCGCCATGAGTAAGGGAGAGGAACTTTTCAC
	cvd2020-03-07d	gacctttcgtctctGTCTcaCAACTCCCGTGAAAAGTTCCTCTCCCTTAC
GFPN-039	cvd2020-03-07e	cgatgtacgtctcaCTCGCCATGAGTAAAGGAGAGGAGCTTTTCACAG
	cvd2020-03-07f	gacctttcgtctctGTCTcaCAACTCCTGTGAAAAGCTCCTCTCCTTTAC

GFPN-040	cvd2020-03-07g	cgatgtacgtctcaCTCGCCATGAGCAAAGGAGAAGAGTTATTTACAGG
	cvd2020-03-07h	gacctttcgtctctGTCTcaCAACCCCTGTAAATAACTCTTCTCCTTTGC
GFPN-041	cvd2020-03-07i	cgatgtacgtctcaCTCGCCATGAGCAAAGGAGAGGAATTATTTACG
	cvd2020-03-07j	gacctttcgtctctGTCTcaCAACGCCCGTAAATAATTCCTCTCCTTTGC
GFPN-042	cvd2020-05-15a	cgatgtacgtctcaCTCGCCATGAGTAAAGGGGAGGAACTCTTTACTG
	cvd2020-05-15b	gacctttcgtctctGTCTcaCAACACCAGTAAAGAGTTCCTCCCCTTTAC
GFPN-043	cvd2020-05-15c	cgatgtacgtctcaCTCGCCATGTCGAAAGGTGAAGAACTTTTCACTG
	cvd2020-05-15d	gacctttcgtctctGTCTcaCAACACCAGTGAAAAGTTCTTCACCTTTCG
GFPN-044	cvd2020-05-15e	cgatgtacgtctcaCTCGCCATGAGCAAGGGAGAAGAGCTGTTCACTG
	cvd2020-05-15f	gacctttcgtctctGTCTcaCAACGCCAGTGAACAGCTCTTCTCCC
GFPN-045	cvd2020-05-15g	cgatgtacgtctcaCTCGCCATGAGTAAGGGTGAGGAGTTATTCACG
	cvd2020-05-15h	gacctttcgtctctGTCTcaCAACGCCCGTGAATAACTCCTCACCCTTAC
GFPN-046	cvd2020-05-15i	cgatgtacgtctcaCTCGCCATGTCTAAAGGAGAAGAACTCTTCACAGG
	cvd2020-05-15j	gacctttcgtctctGTCTcaCAACCCCTGTGAAGAGTTCTTCTCCTTTAG
GFPN-047	cvd2020-05-15k	cgatgtacgtctcaCTCGCCATGTCCAAAGGAGAAGAACTATTCACC
	cvd2020-05-15l	gacctttcgtctctGTCTcaCAACTCCGGTGAATAGTTCTTCTCCTTTGG
GFPN-048	cvd2020-05-15m	cgatgtacgtctcaCTCGCCATGAGCAAAGGAGAAGAACTATTCACG
	cvd2020-05-15n	gacctttcgtctctGTCTcaCAACTCCCGTGAATAGTTCTTCTCCTTTGC
GFPN-049	cvd2020-05-15o	cgatgtacgtctcaCTCGCCATGTCGAAGGGAGAAGAATTATTTACGG
	cvd2020-05-15p	gacctttcgtctctGTCTcaCAACTCCCGTAAATAATTCTTCTCCCTTCG

Table C. Oligonucleotide primer pairs for constructing TIsigner variants of luciferase. The sequences of each forward and reverse primer pair used for constructing each of the luciferase TIsigner variants is shown. The start codon in each of the forward primers is shaded yellow. BsmBI recognition sites (used for Golden Gate assembly into the MIDAS pML1 vector) are underlined. The MIDAS prefix [CCAT] and suffix [AGGA] (reverse-complement = TCCT) for the RLucN modules are highlighted in bold blue and red, respectively.

RLucN TIsigner ID	Oligonucleotide Primer Pair	Primer Sequences (5' to 3')
RLucN-TI-002	cvd2019-06-14c	cgatgtacgtctcaCTCG**CCAT**GACATCAAAAGTATACGACCCAGAG
	cvd2019-06-14d	gacctttcgtctctGTCTca**TCCT**CTGCTCTGGGTCGTATACTTTTGATG
RLucN-TI-003	cvd2019-06-14e	cgatgtacgtctcaCTCG**CCAT**GACAAGTAAAGTTTATGACCCAGAGC
	cvd2019-06-14f	gacctttcgtctctGTCTca**TCCT**CTGCTCTGGGTCATAAACTTTACTTG
RLucN-TI-004	cvd2019-06-14g	cgatgtacgtctcaCTCG**CCAT**GACCAGCAAAGTTTATGACCCAGAG
	cvd2019-06-14h	gacctttcgtctctGTCTca**TCCT**CTGCTCTGGGTCATAAACTTTGCTG
RLucN-TI-005	cvd2019-06-14i	cgatgtacgtctcaCTCG**CCAT**GACAAGCAAAGTTTATGACCCAGAGC
	cvd2019-06-14j	gacctttcgtctctGTCTca**TCCT**CTGCTCTGGGTCATAAACTTTGC
RLucN-TI-006	cvd2019-06-14k	cgatgtacgtctcaCTCG**CCAT**GACTTCGAAAGTTTATGATCCAGAACAG
	cvd2019-06-14l	gacctttcgtctctGTCTca**TCCT**CTGTTCTGGATCATAAACTTTCGAAG
RLucN-TI-007	cvd2019-06-14m	cgatgtacgtctcaCTCG**CCAT**GACATCAAAAGTTTATGATCCAGAACAAAG
	cvd2019-06-14n	gacctttcgtctctGTCTca**TCCT**TTGTTCTGGATCATAAACTTTTGATGTC
RLucN-TI-008	cvd2019-06-14o	cgatgtacgtctcaCTCG**CCAT**GACGTCGAAAGTTTACGATCCAG
	cvd2019-06-14p	gacctttcgtctctGTCTca**TCCT**TTGTTCTGGATCGTAAACTTTCGACG
RLucN-TI-009	cvd2019-06-14q	cgatgtacgtctcaCTCG**CCAT**GACATCGAAAGTTTACGATCCAGAAC
	cvd2019-06-14r	gacctttcgtctctGTCTca**TCCT**TTGTTCTGGATCGTAAACTTTCGATG
RLucN-TI-010	cvd2019-06-14s	cgatgtacgtctcaCTCG**CCAT**GACCTCGAAAGTTTATGACCCAGAAC
	cvd2019-06-14t	gacctttcgtctctGTCTca**TCCT**TTGTTCTGGGTCATAAACTTTCGAG

References

1. Bernhart SH, Mückstein U, Hofacker IL. RNA Accessibility in cubic time. Algorithms Mol Biol. 2011;6: 3.

2. Terai G, Asai K. Improving the prediction accuracy of protein abundance in *Escherichia coli* using mRNA accessibility. Nucleic Acids Res. 2020;48: e81.

3. Kiryu H, Terai G, Imamura O, Yoneyama H, Suzuki K, Asai K. A detailed investigation of accessibilities around target sites of siRNAs and miRNAs. Bioinformatics. 2011;27.

4. Do CB, Woods DA, Batzoglou S. CONTRAfold: RNA secondary structure prediction without physics-based models. Bioinformatics. 2006;22.

5. Bhattacharyya S, Jacobs WM, Adkar BV, Yan J, Zhang W, Shakhnovich EI. Accessibility of the Shine-Dalgarno Sequence Dictates N-Terminal Codon Bias in *E. coli*. Mol Cell. 2018;70.

6. Dirks RM, Pierce NA. An algorithm for computing nucleic acid base-pairing probabilities including pseudoknots. J Comput Chem. 2004;25: 1295–1304.

7. Nieuwkoop T, Claassens NJ, van der Oost J. Improved protein production and codon optimization analyses in *Escherichia coli* by bicistronic design. Microb Biotechnol. 2019;12: 173–179.

8. Pelletier J, Sonenberg N. The involvement of mRNA secondary structure in protein synthesis. Biochemistry and Cell Biology. 1987. pp. 576–581.

9. Voges D, Watzele M, Nemetz C, Wizemann S, Buchberger B. Analyzing and enhancing mRNA translational efficiency in an *Escherichia coli* in vitro expression system. Biochemical and Biophysical Research Communications. 2004. pp. 601–614.

10. Scherr M, Rossi JJ, Sczakiel G, Patzel V. RNA accessibility prediction: a theoretical approach is consistent with experimental studies in cell extracts. Nucleic Acids Res. 2000;28: 2455–2461.

11. van Dolleweerd CJ, Kessans SA, Van de Bittner KC, Bustamante LY, Bundela R, Scott B, et al. MIDAS: A Modular DNA Assembly System for Synthetic Biology. ACS Synth Biol. 2018;7: 1018–1029.
