# Supplementary material for: Analysis of 11,430 recombinant protein production experiments reveals that protein yield is tunable by synonymous codon changes of translation initiation sites
Source: PLoS Comput Biol. 2021 Oct 5;17(10):e1009461. doi: 10.1371/journal.pcbi.1009461 (PMC8519471; doi:10.1371/journal.pcbi.1009461)
Supplement: Attachment — Submitted filename: Response_TIsigner_.pdf [file pcbi.1009461.s014.pdf]

Point-By-Point Responses

Reviewer #1: In this manuscript, the authors use calculations based on an RNA energy model to predict recombinant protein expression levels. They show that by choosing an optimal sub-sequence in the vicinity of the start codon, the mRNA "opening energy" predicted by the Vienna RNA structure prediction package correlates strongly with experimentally determined protein expression levels. After demonstrating that this metric performs well across a variety of existing and new datasets, they construct a simulated annealing algorithm to optimize a codon sequence in order to minimize the opening energy. The authors suggest that this algorithm can be used to tune recombinant protein expression levels and generally outperforms existing codon-optimization software.

Although incorporating analyses of a variety of types of datasets strengthens the results overall, I have the following major concerns about the manuscript:

1. The authors do not define the "opening energy" explicitly. It is not sufficient to report the command-line parameters used in the Vienna RNA package, since this requires a reader to study that software package in order to understand the central message of this paper. A reader needs to be able to understand both the physical meaning of this quantity and how it could be determined, at least in principle, from an experiment or using an alternative RNA structure prediction package.

We have added a more rigorous definition of “opening energy” as well as tools which can compute this quantity in the section **Sequence feature analysis, Methods**, as follows:

“In this study, we use the minimum free energy (MFE), opening energy and avoidance as thermodynamic features of a mRNA. The minimum free energy of a sequence is the energetically most optimal sequence in a Boltzmann’s ensemble. Consequently this feature is based on a single structure. In contrast, the opening energy of a stretch $i..j$ of nucleotides is the pseudo energy required to unpair that region and is given by:

$$Opening\ energy \ = \ -\frac{1}{\beta} ln \frac{Z_{unpaired}}{Z}$$

where the thermodynamic beta $\beta = \frac{1}{kT}$ where k is the Boltzmann’s constant and T is the absolute temperature, $Z_{unpaired}$ is the canonical partition function of the region $i..j$ and Z is the total canonical partition function. The ratio $\frac{Z_{unpaired}}{Z}$ is the probability that the nucleotides $i..j$ are unpaired.

A crucial difference of opening energy with MFE is that $Z_{unpaired}$ captures all possible optimal and suboptimal structures in the Boltzmann’s ensemble, where the nucleotides $i..j$ are unpaired. Many different RNA structures are taken into account with this measure, not just a single optimal structure.

The avoidance measures the number of potential misinteractions, and is calculated by computing the possible interactions among the RNAs. The conventional way to model interactions is to assume a two step process where nucleotides unpair and then hybridise. Thus the total interaction energy ΔG_{int} is given by :

$$\Delta G_{int} = \Delta G_u + \Delta G_h$$

where ΔG_u is the unpairing energy and ΔG_h is the hybridsation energy.

The computation of the partition function and related energetic quantities involve dynamic programming. The implementation of these dynamic programming algorithms is available on several tools such as ViennaRNA and IntaRNA [30,31].“

2. The authors make a point of comparing predictions based on the opening energy, which appears (see point 1) to describe a reversible work or free-energy difference, with predictions based on the MFE structure. First, this idea is not new. For example, reference 68---which the authors claim uses MFE structures---in fact utilizes both free-energy differences and base-pairing probabilities of secondary structure ensembles. Second, the authors search for the highest correlation by varying the sub-sequences used in the opening energy calculations, but appear to keep the sub-sequence used for MFE calculations fixed at -30:30. It is thus unclear whether the better performance of predictions based on the opening energy is due to the ensemble-averaging or the sub-sequence optimization.

We actually claim that either MFE or the metrics derived from MFE. Ref 68 uses unfolding energy which is derived using Gibb’s free energy (ΔG), not the partition function approach as in our work. That metric is related to base pairing, whereas our metric is related to the probability that a base is unpaired. However, since authors in many other works derive their own metrics using their own approaches, we changed that line as follows:

“This partition function based approach is distinct from previous studies where secondary structure stability was defined using MFE or metrics derived from free energy [22,64–69].”

Secondly, we used -30:30 for MFE calculations based on the results from the factorial experiment of Cambray et al (2018). Cambray et al computed MFE for a number of regions and deduced -30:30 as the optimal region in *Escherichia coli*. Furthermore, MFE is usually calculated for a certain region and is a single value (https://www.tbi.univie.ac.at/RNA/RNAfold.1.html), whereas opening energy calculation gives a matrix of energies for subsequences (https://www.tbi.univie.ac.at/RNA/RNAplfold.1.html). This is important because opening energy immediately gives us the landscape view of energies, from which we can identify the regions with the maximum biologically relevant signals. We have added the following line in **Sequence feature analysis, Methods**:
“Based on Cambray and colleagues’ findings, we calculated MFE using the mRNA region −30:30 [19].”

3. The authors claim that their analysis shows that translation initiation, and the accessibility of the Shine--Dalgarno sequence in particular, are key determinants of protein abundance. However, other than the fact that the sub-sequences considered in the opening energy calculations are located near the start codon, nothing in the analysis particularly points to translation initiation or the Shine--Dalgarno sequence. While it is likely that this hypothesis is correct, in my view the evidence directly supporting this hypothesis that exists in the preexisting literature is considerably stronger than that presented here.

Subsequences considered in the opening energy calculations (−30:18 and -24: 24) are actually a superset of the Shine-Dalgarno region -13:-8. Hence we think this might be because the upstream region must be unpaired for an easy recruitment of ribosomes and a successful translation. To clarify this point, we have revised the section **Accessibility of translation initiation sites strongly correlates with protein abundance, Results**, as follows:

"Interestingly, our findings in E. coli also suggest that the surrounding region of initiation sites, including the Shine-Dalgarno sequence [71] at −13:−8, should be accessible, presumably in order to recruit ribosomes. In contrast, the Shine-Dalgarno sequence is absent in yeasts and complex eukaryotes, which may explain why the computed accessibility regions are less than 10 nucleotides preceding initiation codons. In eukaryotes, the 43S preinitiation complex scans from the 5′-cap end of the mRNAs [72]. This mechanism employs helicases such that the RNA structures preceding initiation codons are scanned through.

Theoretically, bacterial 30S subunits can initiate at any position as none-AUG initiation codons are more common than for eukaryotes, and most bacterial mRNAs are polycistronic. However, in agreement with previous high-throughput, RNA structural probing studies, the regions −30:30 of bacterial mRNAs are significantly less structural and are A-rich [73–75]. We reasoned that accessibility is likely more important in bacteria than eukaryotes (Fig 1, stronger correlation between accessibility and protein abundance). High accessibility of initiation sites likely improves a greater selectivity in translation initiation in bacteria."

4. The authors do not provide much in the way of a mechanistic interpretation of their opening energy results. For example, different optimal sub-sequences, which have substantially different average opening energies, are identified for different datasets. Why is this the case, and are the magnitudes of these free energies physically reasonable? Is the opening energy threshold identified in Figure S12 physically meaningful? In addition, many of the figures lack units for the opening energy.

We added an interpretation of opening energy (response to first comment). The partition function used to compute opening energy can be quite different for the same subsequence of different sequences. This is due to a cascading effect in the recursive equations used to compute partition function so that even a few nucleotide differences is likely to have a noticeable impact on the final opening energy of that subsequence. This effect is much stronger if we compare multiple datasets due to many differences in sequences. Furthermore, we also note that because the ratio of partition function, instead of just the partition function, is used for the computation of opening energy, the obtained opening energies are actually pseudo energies. Hence, these quantities may not be reasonable or even realistic in a strictly experimental setting. Nevertheless, they are quite useful in interpreting the unpairedness of mRNA and can be used to computationally design sequences. We have clarified this point in the interpretation of opening energy by referring to it as pseudo energy.

Figure S12, is a logistic regression fitted to the opening energy of sequences from PSI:Biology dataset. Again, since opening energy is a pseudo energy, it may not be

physically meaningful. However, it serves as a useful computational guideline in designing sequences with weak or strong pairing in the desired regions.

We have fixed the units of opening energy in our figures.

The following comments pertain to less significant issues:

1. The description of the simulations as "coarse-grained," and the contrast with ab initio and Molecular Dynamics methods, is a bit misleading. The simulations are more appropriately described as phenomenological kinetic modeling.

We replaced “coarse-grained” with stochastic.

2. The p-values need to be calculated in a way that is not limited by machine precision. Currently, the reported statistical significance is the same for the MFE and opening energy-based approaches (e.g., on page 7).

We note that *PLOS Computational Biology* has no apparent policies regarding such P-value reporting. We further note, that as detailed in Huber (2019) there are good reasons not to report exact P-values: “As scientists, we should not report a p value that is smaller than the probability that there was an unforeseen mishap in the experiment or the processing of the data. Statistical tests and p values are designed to take into account various sources of noise and error in the data and to quantify the associated probabilities faithfully; but they cannot take into account everything” (https://doi.org/10.1016/j.cels.2019.03.001).

3. When performing the sequence optimization by modifying only the first ~9 codons, is simulated annealing absolutely necessary? Have the authors compared the total number of opening energy calculations performed in the course of a complete simulated annealing calculation versus the total number of distinct synonymous sequences? It would be helpful to report some computational performance characteristics, and more explicitly describe the simulated annealing temperature schedule, in order to compare the algorithm with existing approaches.

While using simulated annealing is not absolutely necessary, it saves us time to manually make these codon substitutions by referring to the codon table.

For example, for the following sequence (the major capsid gene of bacteriophage T7 from the below response to Reviewer 2), where the expression score is 88.71 and opening energy is 9.05 kcal/mol:

```
ATGGCTAGCATGACTGGTGGACAGCAAATGGGTACTAACCAAGGTAAAGGTGTAGTTGCTGCTGGAGAT
AAACTGGCGTTGTTCTTGAAGGTATTTGGCGGTGAAGTCCTGACTGCGTTCGCTCGTACCTCCGTGACC
ACTTCTCGCCACATGGTACGTTCCATCTCCAGCGGTAAATCCGCTCAGTTCCCTGTTCTGGGTCGCACT
CAGGCAGCGTATCTGGCTCCGGGCGAGAACCTCGACGATAAACGTAAGGACATCAAACACACCGAGAAG
GTAATCACCATTGACGGTCTCCTGACGGCTGACGTTCTGATTTATGATATTGAGGACGCGATGAACCAC
TACGACGTTCGCTCTGAGTATACCTCTCAGTTGGGTGAATCTCTGGCGATGGCTGCGGATGGTGCGGTT
CTGGCTGAGATTGCCGGTCTGTGTAACGTGGAAAGCAAATATAATGAGAACATCGAGGGCTTAGGTACT
GCTACCGTAATTGAGACCACTCAGAACAAGGCCGCACTTACCGACCAAGTTGCGCTGGGTAAGGAGATT
ATTGCGGCTCTGACTAAGGCTCGTGCGGCTCTGACCAAGAACTATGTTCCGGCTGCTGACCGTGTGTTC
```

TACTGTGACCCAGATAGCTACTCTGCGATTCTGGCAGCACTGATGCCGAACGCAGCAAACTACGCTGCT
CTGATTGACCCTGAGAAGGGTTCTATCCGCAACGTTATGGGCTTTGAGGTTGTAGAAGTTCCGCACCTC
ACCGCTGGTGGTGCTGGTACCGCTCGTGAGGGCACTACTGGTCAGAAGCACGTCTTCCCTGCCAATAAA
GGTGAGGGTAATGTCAAGGTTGCTAAGGACAACGTTATCGGCCTGTTCATGCACCGCTCTGCGGTAGGT
ACTGTTAAGCTGCGTGACTTGGCTCTGGAGCGCGCTCGCCGTGCTAACTTCCAAGCGGACCAGATTATC
GCTAAGTACGCAATGGGCCACGGTGGTCTTCGCCCAGAAGCTGCTGGTGCAGTGGTTTTCAAAGTGGAG
TAA

A single nucleotide change at the sixth position (T -> A) gives a better solution (expression score 93.73, opening energy 8 kcal/mol):

ATGGCAAGCATGACTGGTGGACAGCAAATGGGTACTAACCAAGGTAAAGGTGTAGTTGCTGCTGGAGAT
AAACTGGCGTTGTTCTTGAAGGTATTTGGCGGTGAAGTCCTGACTGCGTTCGCTCGTACCTCCGTGACC
ACTTCTCGCCACATGGTACGTTCCATCTCCAGCGGTAAATCCGCTCAGTTCCCTGTTCTGGGTCGCACT
CAGGCAGCGTATCTGGCTCCGGGCGAGAACCTCGACGATAAACGTAAGGACATCAAACACACCGAGAAG
GTAATCACCATTGACGGTCTCCTGACGGCTGACGTTCTGATTTATGATATTGAGGACGCGATGAACCAC
TACGACGTTCGCTCTGAGTATACCTCTCAGTTGGGTGAATCTCTGGCGATGGCTGCGGATGGTGCGGTT
CTGGCTGAGATTGCCGGTCTGTGTAACGTGGAAAGCAAATATAATGAGAACATCGAGGGCTTAGGTACT
GCTACCGTAATTGAGACCACTCAGAACAAGGCCGCACTTACCGACCAAGTTGCGCTGGGTAAGGAGATT
ATTGCGGCTCTGACTAAGGCTCGTGCGGCTCTGACCAAGAACTATGTTCCGGCTGCTGACCGTGTGTTC
TACTGTGACCCAGATAGCTACTCTGCGATTCTGGCAGCACTGATGCCGAACGCAGCAAACTACGCTGCT
CTGATTGACCCTGAGAAGGGTTCTATCCGCAACGTTATGGGCTTTGAGGTTGTAGAAGTTCCGCACCTC
ACCGCTGGTGGTGCTGGTACCGCTCGTGAGGGCACTACTGGTCAGAAGCACGTCTTCCCTGCCAATAAA
GGTGAGGGTAATGTCAAGGTTGCTAAGGACAACGTTATCGGCCTGTTCATGCACCGCTCTGCGGTAGGT
ACTGTTAAGCTGCGTGACTTGGCTCTGGAGCGCGCTCGCCGTGCTAACTTCCAAGCGGACCAGATTATC
GCTAAGTACGCAATGGGCCACGGTGGTCTTCGCCCAGAAGCTGCTGGTGCAGTGGTTTTCAAAGTGGAG
TAA

Doing this task by hand would take at least 20 random tries, whereas our simulated annealing algorithm can find this solution after a maximum of 10 iterations. In a more general case, for 9 codons, there are a total 19,682 unique synonymous sequences. We have explained this at the first paragraph of **Simulated annealing, Methods**:

“For example, for a protein-coding sequence of nine codons, assuming an average of 3 synonymous codons per amino acid, we can expect a total of 19,682 unique synonymous coding sequences. This number increases rapidly with increasing numbers of codons.”

This justifies the need for simulated annealing, where we can find an optimal sequence, usually after 10-20 iterations for such a large search space. The cooling schedule is exponentially decreasing as presented on supplementary figure 10. We clarified this on the following line at the same section:

“As the temperature cools (exponentially decreasing), the moves get milder with fewer synonymous codon changes (Supplementary Fig S10).“

Reviewer #2: Let me preface my review by saying that I'm a computational biologist and cannot judge the experimental validity of the work presented. The experiments seem reasonable to me, but there may be subtle details that I'm missing.

On the theoretical/computational side, though, I feel authors' thinking lacks in precision and in scholarship. This is obvious throughout the introduction and the general setup of the argument. For example, "Codon usage bias is thought to correlate with tRNA abundance, translation efficiency and protein production." Well, codon usage bias definitely correlates with tRNA abundance. There is no doubt about that. Whether it correlates with translation efficiency and/or protein production depends on many factors, most importantly the specific organism under consideration. For example, here is a study where codon usage is directly linked to protein production in a bacteriophage: https://www.ncbi.nlm.nih.gov/pmc/articles/PMC6938266/ The codon modification explicitly excluded the region near the translation initiation site, so it's a clean and controlled study. Similarly, "but its usefulness has been questioned". Usefulness for what? The sentence seems to imply that any sort of
codon-usage bias is useless, which is an overly broad statement. And let's not forget that there are multiple different causes for codon usage bias. For example, GC content bias causes codon usage bias, but I don't think the authors are thinking about this when they are talking about codon usage bias.

We have revised our introduction as per suggested. We removed the following sentence from our introduction:
“Codon usage bias is thought to correlate with tRNA abundance, translation efficiency and protein production [11–17] but its usefulness has been questioned [18–21].”

We have added the following sentence in Introduction:
“Many mRNAof these features (e.g. CAI, C+G, MFE) are interrelated, which makes the identification of useful features more challenging.”

This point is further explained in the section **Accessibility is a single sequence feature that explains most of the variation in protein abundance, Discussion.** In this section, we have analysed the proteomics dataset (Jack *et al.* 2017, https://doi.org/10.1534/g3.117.041020).

We found the at the dataset compared only two sequences, one with wild-type CAI, the other with low CAI or “codon deoptimisation” that did not account for other interrelated mRNA features. Possibly, Jack *et al.* (2017) assumed that by keeping the first 14 codons intact would avoid influences from other features and make the data “clean and controlled”. In fact, we have found further evidence that opening energy also explains why the low CAI sequence was poorly expressed (see Fig S13 or below). This variance could neither be explained by the traditional MFE approach nor the ‘ramp hypothesis’, due to no change at the first 14 codons.

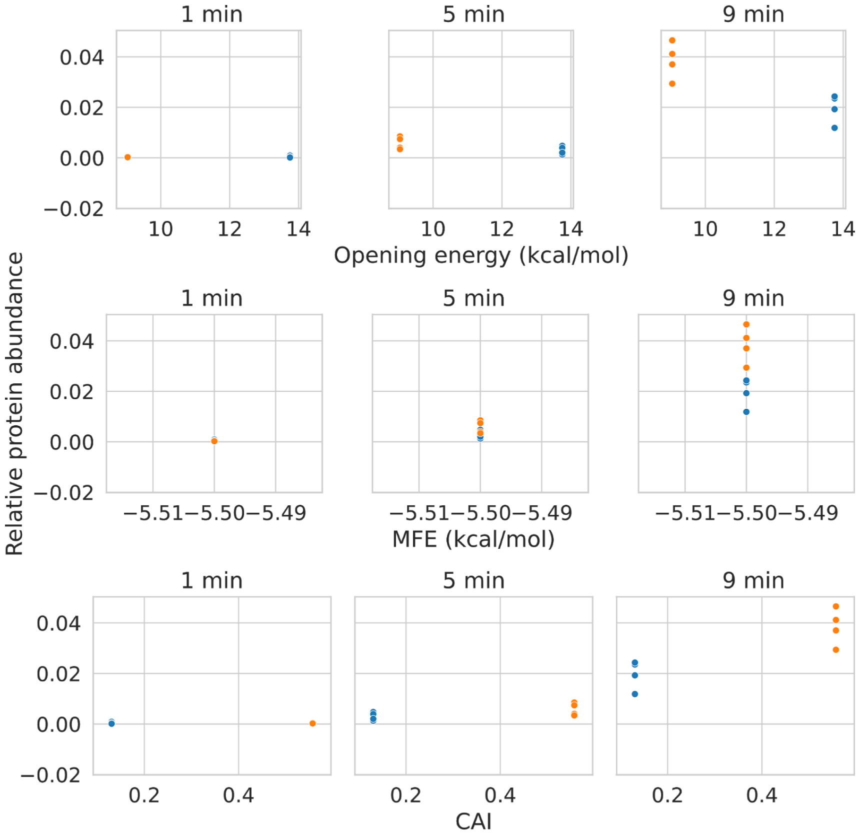

Fig S13. Accessibility is able to capture the full ensemble average energy of a sequence. The levels of major capsid protein expressed by the wild-type (orange) and mutant (blue) strains of bacteriophage T7 [10]. The mutant major capsid gene was codon-deoptimised such that the first and the last 14 codons remained unchanged [10]. Proteomic analyses were carried out at 1, 5 and 9 min post-infection in four biological replicates [10]. Opening energies −24:24, MFEs −30:30, and CAI of the wild-type and mutant sequences were compared. The approximated 'Expression Scores' of the wild-type and mutant sequences are 89 and 38, respectively (opening energies of 9.05 kcal/mol and 13.76 kcal/mol, respectively). MFE, Minimum Free Energy; CAI, Codon Adaptation Index.

At a minimum, we need to distinguish between viruses, bacteria, and eukaryotes. The authors gloss over these issues. The paper seems to be primarily about E. coli, but then the results also talk about yeast and mice.

We agree that our paper is based primarily on *E. coli* due to the lack of recombinant protein expression datasets available to other hosts. However, we also did further analysis on yeast and mice to show that that our approach is generalisable across species. We have modified the manuscript in order to make it clearer that these species are distinguished. See our response to Reviewer 1, comment 3.

I feel that overall, the authors need to put a bit more effort into reading the literature, and then they can carefully rethink how they want to present their argument. Here is a very brief reading list I would suggest as a starting point. There are many more relevant papers:
https://science.sciencemag.org/content/342/6157/475.abstract
https://www.embopress.org/doi/full/10.1038/msb.2010.59
https://journals.plos.org/ploscompbiol/article?id=10.1371/journal.pcbi.1000664

https://www.ncbi.nlm.nih.gov/pmc/articles/PMC5303271/

We thank the reviewer for the suggested readings. We in fact cited and discussed much of the primary literature that these references refer to, together with many additional references with the 118 references provided with the original manuscript. We regret that in a research article, we are unable to cite all the existing literature that is of some relevance to the current study. Perhaps in future, a review article could be produced that may do so.

Reviewer #3: In this manuscript, Bhandari et al address an important problem of optimizing expression of recombinant proteins in different kinds of cellular expression systems. Using a large collection of existing protein expression databases and computational prediction of RNA structures, the authors show that accessibility of translation initiation sites in the mRNA is the best predictor of expression levels, in contrast to minimum free energy (MFE) which is the commonly used parameter. They develop a simulated annealing-based method to modify sequences using synonymous substitutions which can optimize opening energies at the translation initiation sites. This work is important for those who wish to optimize expression of foreign proteins, and in my opinion, TIsigner program that the authors develop to optimize synonymous codons in the first 9 codons of a transcript is the major strength of the work. However, I do have some major concerns mostly regarding the novelty of the work that needs to be addressed.

1. It is well-known from earlier studies (Bhattacharyya et al, Mol Cell, 2018; Goodman, Science, 2013; Kudla et al, Science, 2009 and others) that mRNA secondary structures that prevent translation initiation is the predominant factor that dictates protein expression. Some of these studies have specifically shown that the RBS (Shine-Dalgarno region) needs to be free of structure. In the light of these, I am not entirely clear about the novelty of the results presented here. The authors coin the term opening energy, but I do not see it defined anywhere properly in terms of energetics. Is it the energy required to unfold a certain length of the mRNA? I presume it is related to the probability of a particular region of the N-terminus to be unbound in the ensemble of predicted secondary structures. Also, the authors do not clearly define what they call translation initiation site. I presume it is the start codon (AUG).

We added a rigorous explanation of opening energy (see response to Reviewer 1 first comment). Translation initiation site is usually regarded as the start codon AUG. We clarified this in multiple places in this revision.

How different will the correlations be if the authors were to look only at accessibility of the SD or an equivalent region in case of eukaryotic sequences? If the authors show that accessibility of the start codon is superior to explain protein abundances than that of SD, it would be a novel extension of known understandings, in my opinion.

We thank the reviewer for this curious insight. We presented the correlation heatmaps between the accessibility and protein expression that encompass all possible subsequences in Fig 2 and Supplementary Fig S6. Given that an SD sequence is located at −13:−8, the

way to pinpoint this region in the heatmap is to check for the point $i = -8$ (x-axis) and $l = 21$ (y-axis). Similarly, for the start AUG, we can look at the point $i = 3,\ l = 3$. Interestingly, in Fig 2B, C, and E, both of these points have a lower correlation and the Area Under the ROC Curve than the region that we identified to have a stronger correlation (−24:24). This suggests that a slightly larger region around those key motifs provides a better context of accessibility and thus a better prediction of protein expression. A good example is illustrated in our above response to reviewer 2, in which by keeping the first and the last 14 codons intact for "codon deoptimization", the accessibility of the initiation site (−24:24) can still be affected. This example highlights the novelty of our methods.

2. Some of the studies that the authors cite for MFE actually use an ensemble of structures and not the MFE structure. This should be made clear.

We have made changes to address this issue (see response to Reviewer 1, comment 2).

3. Why is that the region of the mRNA whose opening energy most significantly correlates with expression so different in different expression systems? -30:18 for E. coli, -7:89 for yeast and -8:11 for Mus Musculus. Any insight will be helpful

We have addressed this question in response to Reviewer 1, comment 3. We have explained the key distinctions between bacterial and eukaryotic translation initiation mechanisms that may be related to these observations. We are likely to get different regions across different species possibly due to the differences in their translation machinery and process.

Minor:

1. Fig 3C: this matrix is unclear. Is protein expression correlated to some binary parameter here? If so, how are they combined? Many of these are not independent parameters. Similar question with Fig 4A.

The dataset used to calculate this matrix had a binary label for whether the protein was expressed or not. This matrix is the correlation between the features and this binary label. We modified the caption to address this.

2. Fig 4A, the data points for luciferase alone do not seem to anti-correlate with opening energies (just eyeballing). Any reason why that might be?

This could be due to protein aggregation, as the results in the SDS-PAGE and luciferase reporter analyses are slightly different. See Supplementary Fig S14 and the section **Low protein yields can be improved by synonymous codon changes in the vicinity of translation initiation sites, Results**:

"Additionally, we tested this finding using the luciferase reporter from *Renilla reniformis* (RLuc). We designed and tested a series of nine RLuc variants with higher accessibility than the wild-type sequence, in which sequence optimisations were performed within the first 9 codons (opening energies of 5.77-10.38 kcal/mol; Supplementary Fig S14 and Table S5).

We also tested five commercially designed sequences, which incorporated sequence optimisations across the entire RLuc gene. The TIsigner optimal sequence (5.77 kcal/mol) was expressed at a higher level compared to a TIsigner suboptimal sequence (10.38 kcal/mol) and the wild type (13.15 kcal/mol) in the BL21Star(DE3) *E. coli* host. However, it is worth noting that a high proportion of the expressed RLuc protein is insoluble, as analysed by SDS-PAGE (Supplementary Fig S14A). We also carried out luciferase reporter assay to compare the levels of active soluble RLuc but detected no significant differences between the luminescence levels of these three sequences. The poor correlation between RLuc protein abundance with luciferase activity could be partly attributed to the observed aggregation problems in Supplementary Fig S14A."

3. Right panel of Fig 4B needs better explanation. I presume the inset graphs shows the growth rate data, but this requires some quantification.

We added an explanation for the insets.

4. The supplementary tables are between Fig S5 and S6 for some reason, it will be good to have the figures together and the tables later.

As per PLOS Guidelines, we cited these figures and tables according to the order in the manuscript text so that readers can follow along.

5. Fig S6: why is the green triangle region zoomed in? Why is this region special?

This allows visual comparison at the exact same regions across species.
