# Supplementary material for: Analysis of 11,430 recombinant protein production experiments reveals that protein yield is tunable by synonymous codon changes of translation initiation sites
Source: PLoS Comput Biol. 2021 Oct 5;17(10):e1009461. doi: 10.1371/journal.pcbi.1009461 (PMC8519471; doi:10.1371/journal.pcbi.1009461)
Supplement: Attachment — Submitted filename: Response_r2.pdf [file pcbi.1009461.s015.pdf]

Response to reviewers (round two)

Editor's comments:
The authors addressed several critiques outlined in initial reviews. However Ref 2 raises issuea that are fundamntal to claims of novelty and significance presented in this paper. The authors should have another chance to address them with subsequent re-review by Ref.2.

We have addressed these issues point-by-point as follows, and revised the main text and Supplementary Information accordingly (as blue-coloured text). To improve the readability of the manuscript, we have simplified the Abstract and reordered the main text as Results, Discussion and Methods. We have also reformatted the text according to the PLOS guidelines.

Reviewers' comments:
Reviewer #1: The authors have adequately responded to my review. I have no further concerns.

Thank you.

Reviewer #2: The clarifications provided by the authors, in particular the definition of the unfolding energy, are helpful. However, the changes do not satisfactorily address the concerns laid out in the original review. First, the relationship between the proposed method and existing approaches in the literature is inaccurately described, and the improvement over existing approaches is thus insufficiently demonstrated (original point 2). Second, the mechanistic interpretations are not sufficiently justified (original points 3 and 4). These concerns are described further below.
Concerning point 2, there appears to be confusion regarding the relationship between free energies and partition functions. What the authors call opening energy is the free energy difference between two ensembles of structures, one with partition function Z_unpaired and the other with partition function Z. To clarify, there are two types of free energies that appear in RNA structure calculations. The first is the free energy of a particular structure labeled i, g_i. The MFE is equal to the minimum g_i of the all possible structures considered by the RNA folding model. The second free energy is related to the partition function of an ensemble of structures: G = -kT log Z, where Z = sum_i exp(-g_i/kT). Free energy differences, delta G = G_2 - G_1, between two ensembles of structures with partition functions Z_1 and Z_2 are physically meaningful.
The delta G used in the current ref 64 is also a free energy difference defined between two ensembles of structures, although the definition of these ensembles is slightly different from the unfolding energy used here. Thus, these methods are very closely related. Because of the direct relationship between free energies of structure ensembles and the corresponding partition functions, there is no qualitative distinction between the approach used in this work and those used in some previous studies. The revised text "This partition function based approach is distinct..." is consequently misleading. Furthermore, the idea that calculations using ensembles of RNA structures can predict the probability of specific bases being unpaired is also not new. For example, ref 64 also makes use of the probability that a particular base is unpaired, which is equal to Z_{i unpaired}/Z. In my view, the paper will be

strengthened by accurately describing how the proposed method differs from previously published approaches.

We apologise if we misled the reviewers about the claim of novelty of the accessibility method that we are using. We did not claim that this method is new in the text. We calculated accessibility using RNAplfold developed by Bernhart et al., 2011 (PMC3063221), which we have cited in the text (ref 36, now ref 22). We did not invent accessibility and do not claim that opening energy calculated using the partition function in RNAplfold is superior to the existing methods.

We also apologise for the misleading statement about the distinction between these two methods. We have revised "This partition function based approach is distinct..." to

> "Previous studies have defined 'accessibility' using different terms [14, 24, 28–33] (see Supporting Information, S2 File). Here we use a partition function method implemented in RNAplfold to calculate accessibility [22] (Fig 1, see Methods)."

We did not coin the term 'opening energy' (see the parameter −O, −−opening_energies in the RNAplfold manual https://www.tbi.univie.ac.at/RNA/RNAplfold.1.html). Bernhart et al., 2011 (ref 36, now ref 22) define 'accessibility' as the probability that the bases are unpaired in a thermodynamic equilibrium (p^u) and 'opening energy' as $\delta G_{openen} = -RT log(p^u)$. We have further elaborated these in our next response. Bernhart et al., 2011 (ref 36, now ref 22) derive the exact equation for these quantities in terms of partition functions, including an auxiliary matrix which further reduces the time complexity. Furthermore, they also provide a tool called RNAplfold, which has the implementations to compute δG_{openen}.

Note that we used the output from RNAplfold directly (δG_{openen}) rather than calculating opening energy ourselves. See also the example output from RNAplfold in our response to Reviewer 3. Some authors prefer to call these ensemble free energies "pseudo-energies" http://rna.informatik.uni-freiburg.de/Teaching/index.jsp?toolName=accessibility, https://www.tbi.univie.ac.at/RNA/RNAplfold.1.html. We have cited the paper appropriately (ref 64, now ref 28).

We think that the novelty of our work is largely the implementation of accessibility in a user-friendly web server and the validation using multiple, independent large-scale datasets, and our own experiments. In addition, we note that our stochastic simulation model is, as far as we are aware, unique. We have added the following sentences in the main text to illustrate the contributions of this study:

> "The existing algorithms for gene optimisation sample synonymous protein-coding sequences use models based on CAI, tAI, MFE, and/or G+C content (%) [23–27]. However, these models are usually based on some of the above findings that rely on either endogenous proteins, reporter proteins, or a few heterologous proteins with their synonymous variants. It is unclear whether these features are generalisable to explain the expression of all heterologous proteins. To address this question, we studied multiple large-scale non-redundant recombinant protein production (N=11,430), proteomic (N=3,725), and fluorescent reporter (N=82,002) datasets from bacterial and eukaryotic species in order to identify mRNA features that best explain variation in protein

abundance. This problem has not previously been investigated with this scale of heterogeneous datasets in any previous studies.

We find that mRNA accessibility is the single best predictor of protein expression across datasets, and accurately predicts the success or failure of 11,430 experiments of recombinant protein expression in *E. coli*. Specifically, the accessibility of translation initiation sites appears to outperform other mRNA features by capturing all possible optimal or suboptimal structures beyond translation initiation sites. With this information, we propose how accessibility can be exploited to fine-tune recombinant protein expression at a low cost. To further this cause, we have built a web server called TIsigner (Translation Initiation coding region designer), which optimises a protein-coding sequence by suggesting synonymous codon changes within the first nine codons. Therefore, our approach makes gene optimisation accessible, as PCR can be used rather than an expensive full-length gene synthesis."

Despite the fact that using ensemble calculations and unpairing probabilities is not new, it is quite possible that the precise definitions of the ensembles used by the authors provide better predictions of protein abundance than existing methods. To the best of my knowledge, the particular free energy difference that the authors use here has not been examined elsewhere. If this particular definition is superior to those examined previously, then this is a useful contribution. However, without making a direct comparison between the proposed quantity and the existing ensemble-based methods in the literature, the authors do not make a convincing case for adopting their proposed method.

We would like to reiterate that we did not propose a new method to calculate accessibility, as we use RNAplfold (ref 36, now ref 22) for our analysis. Furthermore we are finding it difficult to access publicly available software that implements the previously described approaches.

We are thankful and agree to the reviewer that benchmarking the existing accessibility computations would be novel and useful. While trying to apply Bhattacharyya et al.'s method (ref 64, now ref 28) to all possible sub-sequences, we estimate that it will take 19919.6 years to analyse the PSI:Biology dataset (N=11,430) on a current, modern CPU. This suggests that this approach is not a practical solution to the problem. Let us explain further:

Given that the length of the T7lac 5′UTR is 71 nt, a fixed 5′-most coding sequence length of 187 nt (~60 codons) for 11,430 sequences - it takes 0.05 second to calculate a correlation coefficient and an AUC score. Therefore, a complete solution will take 33154 * 33154 * 11430 * 0.05/86400/365 = 19919.6 years

If we had access to 19,920 machines (which exceeds the resources available to NZ-based researchers), it would still take one year to run this analysis. The reason why there are 33154 * 33154 calculations per sequence (all possible sub-sequences for 258 nt, i.e, 71 nt + 187 nt) is because to derive $\Delta G_{unfold}(L)$, we have to compute $\Delta G_{1,n} - \Delta G_{L,n}$. A similar complexity problem also applies to other methods.

Bernhart et al., 2011 (ref 36, now ref 22) have optimised RNAplfold to compute accessibility of all sub-sequences and used an auxiliary matrix that vastly reduces the time complexity. It took 10 hours (without parallel computing) to complete the same task using RNAplfold. Therefore, we are currently unable to make a direct comparison using Bhattacharyya et al.'s and other methods without further algorithm and code optimisation.

Furthermore, to the best of our knowledge, the tool RNAplfold used by us has a different underlying algorithm and mathematical basis than alternative methods, because Bernhart et al., 2011 (ref 36, now ref 22) derive an exact solution for base-unpairing. To avoid confusion, we have expanded the equation of opening energy computation in the main text and listed all the approaches that we know of, which are used to compute the accessibility in Supporting Information (now S2 File) as follows. Note that we used the output from RNAplfold directly (δG_{openen}) rather than deriving opening energy using the expanded equation. See also the example output from RNAplfold in our response to Reviewer 3.

"For a transcript of length n, previous studies have defined 'accessibility' in terms of:

1. Opening energy or base-unpairing probability (as implemented in RNAplfold by Bernhart et al., 2011, used in this paper):

   $\delta G_{openen} = -RT log(p^u)$

   where p^u is the probability that the base pairs between $i...j$ is unpaired and is given by:

   $p^u = \frac{Zunpaired(i...j)}{Z(1,n)}$

   $= \frac{1}{Z(1,n)}(Z(1, i-1)Z(j+1, n) +$

   $\sum_{a \le i} Z^H(a, j) + \sum_{a \le i} Z^I(a, j) + \sum_{a \le i} Z^{M5}(a, j)$

   $+ \sum_{a \le i} Z^{M3}(a, j) + \sum_{a \le i} Z^{MB}(a, j)$

   where, Z^H, Z^I, Z^{M5}, Z^{M3}, Z^{MB} are the partition function contributions from hairpin, interior loop, multiloop contributions at 5` and 3` end and unpaired intervals between the multiloop and the closing base pair. A similar base-unpairing approach is also implemented in Raccess (Terai and Asai, 2020; Kiryu et al., 2011), which further incorporates the stochastic context-free grammar and log-linear models of CONTRAfold (Do et al. 2008).
2. Unfolding energy (Bhattacharyya et al., 2018):

   Unfolding energy for the first L bases is defined as:

   $\Delta G_{unfold}(L) = \Delta G_{1,n} - \Delta G_{L,n}$,

   where, $\Delta G_{1,n}$ is the folding free energy of the entire transcript and $\Delta G_{L,n}$ is the free energy contributions from base-pairings which does not involve the first L bases. These ΔGs are calculated using NUPACK (Dirks and Pierce, 2004).

   <Taken from Bernhart et al., 2011>

   Note that these quantities involve computation of $Z_{base-pairing}$ (https://doi.org/10.1002/jcc.20057), which is not equal to $Z_{unpaired}$ as used in the above approach (Bernhart et al., 2011, ref 36, now ref 22), although they may be

correlated. In addition, pseudoknots are taken into account by NUPACK. The key differences between $Z_{base-pairing}$ and $Z_{unpaired}$ has been illustrated by Bernhart et al., 2011 (ref 36, now ref 22), Fig 1 and 3 shown below.

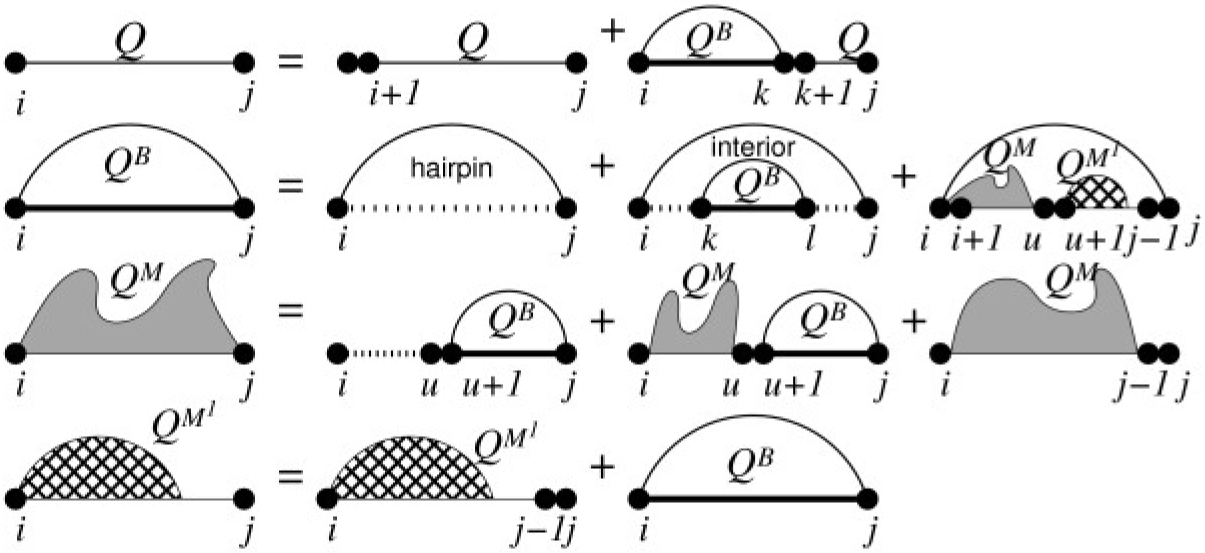

Figure R1 Partition function folding. DP decomposition of RNA partition function folding. See text for detailed explanation. Taken from Bernhart et al., 2011, Figure 1 (ref 36, now ref 22).

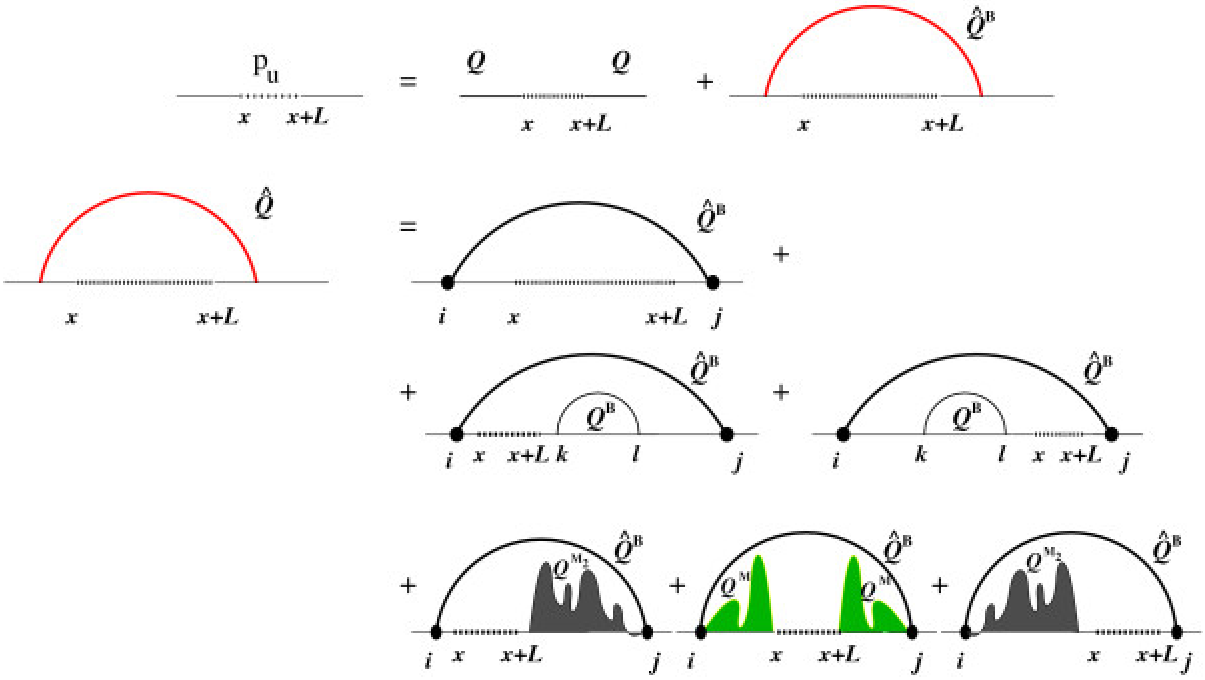

Figure R2 Unpaired interval computation. Diagrams of the probability to be unpaired for an interval of length L starting at base x. The unpaired interval is either enclosed by a base pair or not (top). If enclosed by a base pair, the enclosing base pair can enclose a Hairpin, an Interior Loop or a Multi Loop (rows two to four). Taken from Bernhart et al., 2011, Figure 3 (ref 36, now ref 22).
</>

3. Secondary structure (Nieuwkoop et al., 2019, Pelletier and Sonenberg, 1987):

Transcripts with stable secondary structures (low minimum free energy) are considered less accessible.

4. Base pair probability (Voges et al., 2004):
   Transcripts with higher base pairing probability are less accessible. The base pairing probability is calculated using McCaskill's algorithm, rather than unpairing probability.
5. Structure alignment (Scherr et al., 2000):
   Five sub-sequences of window size (1400 nt) W_{size} with the lowest MFE is aligned. The window is then shifted by 500 nt. The structures of overlapping windows are then further aligned. Repeating this procedure, the accessible motifs were selected only if it is conserved across the windows among several MFE structures.
   Accessibility score SC_{acc} is then defined as:
   $SC_{acc} = [S_i \times P_i \times (W_{s1,i}) / 100]/(W_{seq,i} + W_{tot})$
   where S_i is the average length of predicted target motifs, $W_{s1,i}$ is the proportion of analysed windows with the lowest MFE structure $s1$ with the motif, $W_{seq,i}$ is the number of windows containing the motif and folds into one or more $s1$ structures and W_{tot} is the total number of analysed windows."

We have also added the following paragraph in the Discussion:

"The accessibility of a region of mRNA can be understood as the ability of that region to basepair with other nucleotides, including the flanking region. There are two distinct ways to define the accessibility of a region. (i) The first way is to consider the minimum Gibbs free energy (MFE) of the region. A lower value of MFE implies a stronger folding at that region. Hence, the region is less likely to be available for pairing. The region is then said to be less accessible. Thus, several authors have defined accessibility using the Gibbs free energy, which reflects the strength of mRNA folding around the region of interest [14, 24, 28, 29, 31, 32]. Since, the MFE can be calculated through the computation of partition function, a more rigorous way to define accessibility is to use the partition function to compute the basepair probabilities [30, 76]. Despite the improvements over MFE, this partition function-based approach has not been widely adopted. (ii) Another way that accessibility has been defined is to use the partition function to compute the probabilities of bases being unpaired, or an equivalent pseudo energy called the opening energy [22]. In this work, we use this approach to define accessibility as it is mathematically well-defined and provides greater contextual information (See Fig 1, Fig 4A). Furthermore, an efficient algorithm to compute opening energy is implemented in the RNAplfold of the ViennaRNA suite [77]."

Concerning point 3, the authors' speculation as to why the highest correlation is achieved when unpairing ~50 bases seems plausible. However, the analysis here does not provide specific evidence in direct support of the conclusion that accessibility for translation initiation is the central mechanism at play. For example, convincing evidence might involve showing that the Shine--Dalgarno sequence (in E. coli) or the start codon are specifically implicated by the analysis. Thus, in my view, existing studies in the literature provide much stronger support for the translation-initiation mechanism.

We apologise if we misled the reviewers as our intention is to interpret the data based on models derived from previously published papers, which we have thoroughly cited, rather than making speculations or drawing any conclusions based on the limited datasets for eukaryotes. Our studies show correlations and not causations, as stated in the main text:

> "Taken together, our findings suggest that the accessibility of translation initiation sites strongly correlates with protein abundance across species."

To avoid misunderstanding, we have added the following sentences to explain the caveats:

> "However, caution is in order here, as large-scale recombinant protein production datasets for these eukaryotes are not available to validate these findings. Further investigation into the differences in the mechanisms of translation initiation between prokaryotes and eukaryotes would be useful to explain why these mRNA regions are distinct."

In addition, we would like to apologise if we misled the reviewers regarding the aim of our study. We aim to address the low expression problems of recombinant protein production and provide useful tools for varying this, which we have laid out in the Abstract and Introduction, not the mechanism of translation initiation, and the mechanistic differences between prokaryotes and eukaryotes.

Concerning point 4, free energy differences calculated by RNA structure prediction methods do indeed have physical meanings. As clarified above, free energy differences (not pseudo energies) are related to partition function ratios by Delta G = -kT log (Z_2 / Z_1). Therefore, the interpretation of the free energy difference is that exp(-Delta G / kT) is the relative probability of structure ensembles 1 and 2. Of course, the RNA folding models have been parameterized for solution conditions (e.g., ionic strength and temperature) that may not match physiological conditions, the models make assumptions that restrict the allowed RNA structures, and the influence of the complex intracellular environment is completely neglected; thus, it is likely that the calculated free energy differences are approximate. Using these numbers as proxies for weak versus strong base pairing, as the authors suggest, is thus completely fine.

Nonetheless, the interpretation as the relative probability of structure ensembles becomes important when arguing in favor of a mechanism, such as the translation-initiation mechanism. Specifically, the opening energy is related to the probability that a subset of bases are unpaired. When considering a region of ~100 bases and a free energy difference in excess of 20 kcal/mol (e.g., Figure 1B), one has to ask whether it is physically reasonable for this quantity to be related to the proposed translation initiation mechanism. If the unfolding energy is being proposed solely as a proxy for designing optimal sequences, as the authors now state, then there is no issue with the interpretation of the actual value. However, if the authors want to use these calculations to support an argument in favor of a specific mechanism, as the authors claim elsewhere in the paper, then it is necessary to reconcile the magnitudes of these free energy differences with the proposed mechanism as well as to explain why the magnitudes differ so much from one organism to the next.

As pointed out by Bhattacharyya et al., 2018 (ref 64, now ref 28),

“With this choice of , the predicted typical magnitude of ΔG_{unfold} is a physically reasonable $10\,kT$”.

Using $RT = kT \times N_A$, where N_A is the Avogadro’s constant, we get $\Delta G_{unfold} = 24.79\,kcal\,mol^{-1}$. From our analysis (Fig 1B, now fig 2B), the magnitudes of opening energy are around $25\,kcal\,mol^{-1}$, which is close to the proposed value.

The mRNA region for yeast that shows the best correlation between protein levels and MFE values is -15:50 (ref 17, now ref 19, see Fig 3 as below), which is close to our estimated mRNA region for opening energy (-7:89). As pointed out by Dvir et al., 2013 (ref 17, now ref 19), “Our results suggest that sequence variants that are folded into stable mRNA secondary structures tend to produce less protein (R2 = 0.18; Fig. 5D, light blue panel), most likely because such structures impede translation initiation (21, 34, 35, 45).”

Taking Dvir et al.’s interpretation and the reviewer’s point into account, we have revised our manuscript as follows:

> “However, caution is in order here, as large-scale recombinant protein production datasets for these eukaryotes are not available to validate these findings. Further investigation into the differences in the mechanisms of translation initiation between prokaryotes and eukaryotes would be useful to explain why these mRNA regions are distinct.”

<Taken from Dvir et al., 2013>

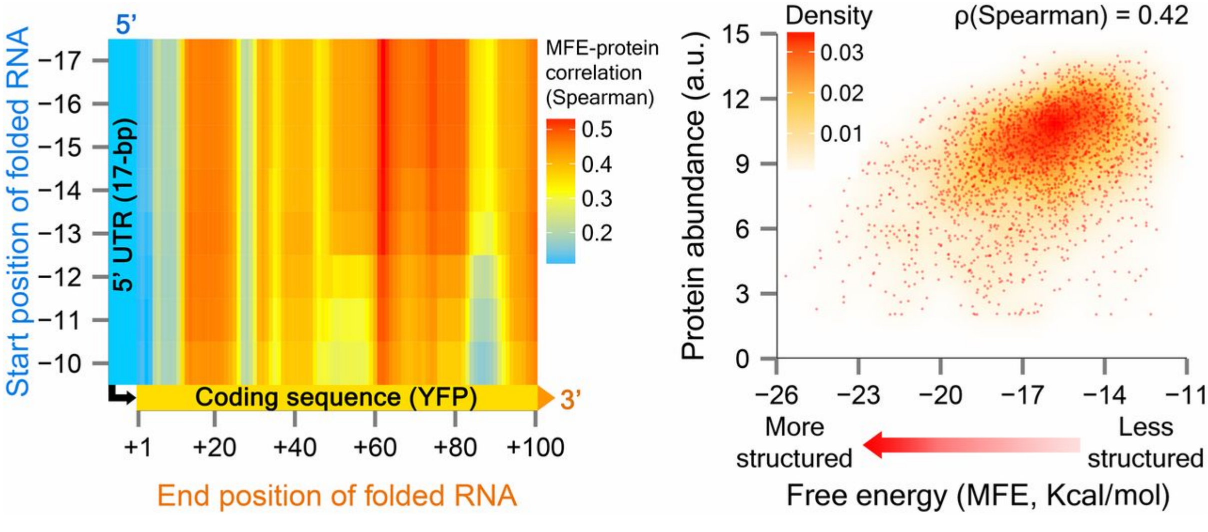

Figure R3. Stable mRNA secondary structures are correlated with reduced protein levels. The left panel shows a heat map of Spearman correlations, where each correlation denotes the association between the minimum free energy (MFE) of a given mRNA region and protein levels, in 2,041 variants. We used the region between −10 and −1 as the smallest folding segment, while moving upstream and/or downstream in 1-bp steps (up to −17 and +100). The y and x axes represent the start (in 5′-UTR) and end (in coding region) position of each folded segment, respectively. For each correlation, we obtained a two-sided P value by performing 100,000 permutation tests of shuffled expression values. For clarity of the figure, we removed folding segments that include only 5′-UTR nucleotides, without the YFP coding region (the Spearman's ρ for these regions is 0.09 ± 0.003; median ± MAD; P < 0.001). The

right panel shows an example of the above relationship for the mRNA segment between positions −15 to +50 (Spearman's $\rho = 0.42$, $P < 10-86$). We obtained equivalent results, albeit with slightly higher correlations, by using the ensemble free energy measure (SI Appendix, Fig. S10, and related SI Appendix, Note S4). Folding free energies were computed with RNAfold from the Vienna RNA package at a folding temperature of 30 °C (51). Taken from Dvir et al., 2013, Figure 3 (ref 17, now ref 19).
</>

Reviewer #3: The authors have addressed several comments, most importantly now provided a detailed procedure of calculating opening energies. Unfortunately, after going through the definition, it seems that 'opening energy' is very similar to the DGunfold parameter, or more importantly the 'punbound' parameter used in reference 68 (now ref 64). This paper also arrived at the same conclusion that accessibility of the SD region is important to dictate mRNA and protein expression. As I mentioned previously, it is well established from several previous work that keeping the 5'-terminus of the mRNA sequence free of structure is important to allow efficient ribosome binding and translation initiation, and this is precisely their finding. Hence, the question about the novelty of the work remains. Additionally, in response to Reviewer 1 the authors say that ref 68 does not use a partition function-based methodology, but that is not correct.

Apologies for this confusion. By "partition function-based methodology", we mean to imply, partition function-based methodology as of RNAplfold. Please see our detailed response to Reviewer 2 (concerning point 2).

As Reviewer 1 also mentions,
the authors should calculate MFE by varying the length of the subsequences, otherwise the comparison between MFE and opening energy remains invalid. I didn't understand what the authors meant while saying that 'MFE is usually calculated for a certain region and is a single value'.

Regarding the comparison between MFE and opening energy, we used the best MFE regions that were previously identified (now ref 16, 19, 36-39). For example, see the subsequence analysis carried out by Dvir et al., 2013 (ref 17, now ref 19) in our response to Reviewer 2 (concerning point 4). As the aim of our study is to improve recombinant protein expression, we have calculated MFE (using NUPACK) for all possible sub-sequences for the PSI:Biology dataset and the respective AUC scores. We obtained the highest AUC scores (0.68) for the mRNA region −28:37, which is within the confidence intervals of the AUC score for the mRNA region −30:30 (Fig 4C, AUC=067, confidence intervals=0.66-0.68).

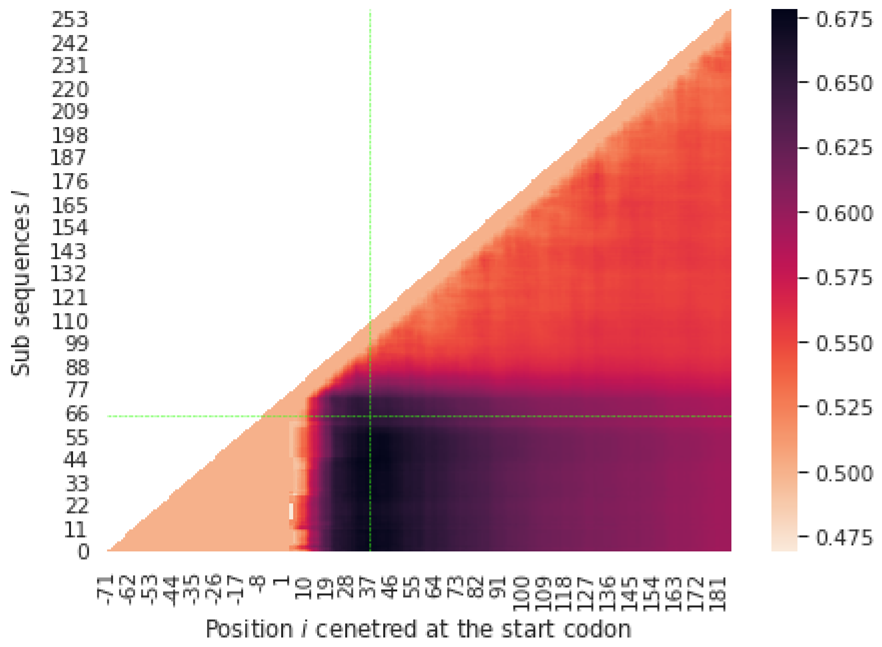

Figure R4. Sub-sequence analysis using NUPACK derived MFE (with default parameters) for the PSI:Biology dataset (N=11,430). AUC scores are shown as a heatmap, in which the highest AUC score (0.68) is indicated by a green crosshair for the region −28:37.

However, there is a significant limitation using the MFE approach, whether or not the mRNA region is selected using a sub-sequence analysis. See the following figure:

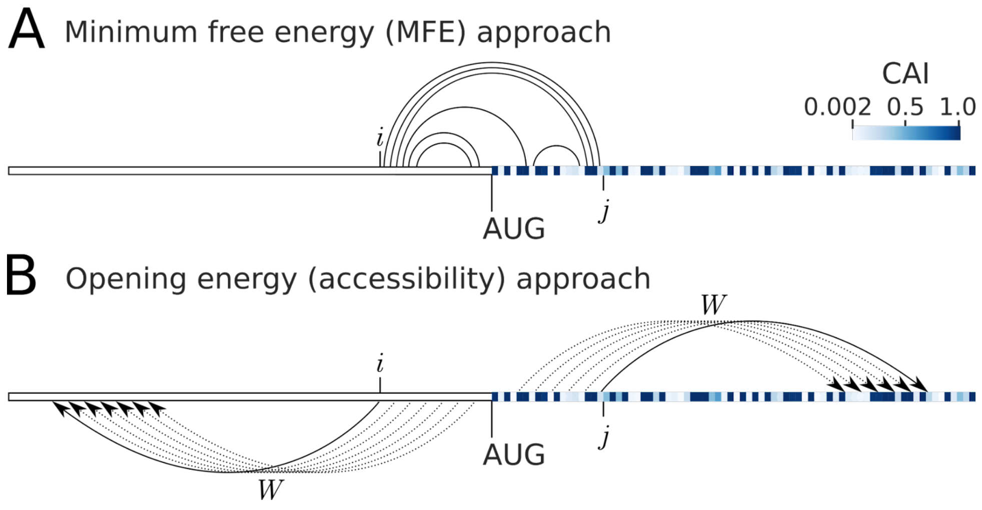

Figure R5. Opening energy (accessibility) has greater contextual information than minimum free energy (MFE). Schematic representation and interpretation of MFE and opening energy of a mRNA sequence (GFP). Codons are color coded using the weights of Codon Adaptation Index (CAI) from Sharp and Li (1987). **(A)** The computation of minimum free energy of the region $i..j$ results in finding a single most stable structure. This stable structure contains pairings within the region as indicated by arcs. Hence, this approach is unable to detect any information beyond the target region (initiation site in this case). For example, a change in CAI after the nucleotide j does not affect the MFE. In addition, the predicted single structure may not be present under physiological conditions. **(B)** The computation of opening energy of the region $i..j$ uses several windows, each of length W

nucleotides, shown by dotted and solid arrows (flanking window). The partition function used to determine the opening energy is computed over all possible structures (optimal and suboptimal) from the Boltzmann's ensemble where the region $i..j$ is unpaired (Material and Methods). As these windows could be extended well beyond the target region, opening energy contains additional contextual information. For example, a change of CAI beyond the nucleotide j could influence the opening energy (Results, Accessibility captures the full ensemble average energy of a sequence). Taken from the current version of our manuscript, Figure 1.

MFE is unlikely to explain the results of experiments where there are no nucleotide changes within the −30:30 region of a mRNA, but with changes beyond −30:30 (e.g., Jack et al., 2017, https://doi.org/10.1534/g3.117.041020), as illustrated in Fig 1A (previously Fig 3A) and our previous response to Reviewer 1 (previously Reviewer 2). We get a constant MFE if there are no nucleotide changes within the −30:30 region, meaning that any nucleotide changes beyond −30:30 would also give rise to a constant MFE (by keeping −30:30 the same and mutating surrounding nucleotides). This is because MFE does not capture the contextual information like opening energy does. See below:

> "**Accessibility captures the full ensemble average energy of a sequence**
> To illustrate the advantage of accessibility over MFE, we analysed a proteomic dataset of E. coli cells infected with bacteriophage T7 [53]. The major capsid gene was codon-deoptimised to generate a mutant T7 strain [53]. Specifically, the first and the last 14 codons of the major capsid gene remained unchanged, making this a good case study to compare accessibility, MFE, and CAI.
>
> We calculated the opening energies, MFEs and CAIs of the wild-type and mutant sequences (Supplementary Fig S13, orange and blue colours, respectively). We also scored the wild-type and mutant sequences using the above logistic regression curve, and obtained the approximated 'Expression Scores' of 89 and 38, respectively (opening energies of 9.05 kcal/mol and 13.76 kcal/mol, respectively). The MFEs of the wild-type and mutant sequences are the same because the local regions −30:30 are identical.
>
> In contrast to MFE, accessibility was able to make an accurate prediction by capturing the full ensemble average energy of the mutant sequence. Importantly, although we use the region −24:24 for the accessibility computation, we are able to capture the key propensity beyond this region. This is because the computation of the partition function, thus the opening energy, for the region −24:24, also utilises the surrounding region (here 210 nucleotides around −24:24, see Methods and Fig 3A4A). This unique approach makes opening energy more robust than MFEthe traditional MFE approach. These results also highlight that mRNA features are interrelated (accessibility and CAI in this case), as such a careful factorial design is necessary to identify the causal features [16,54]."

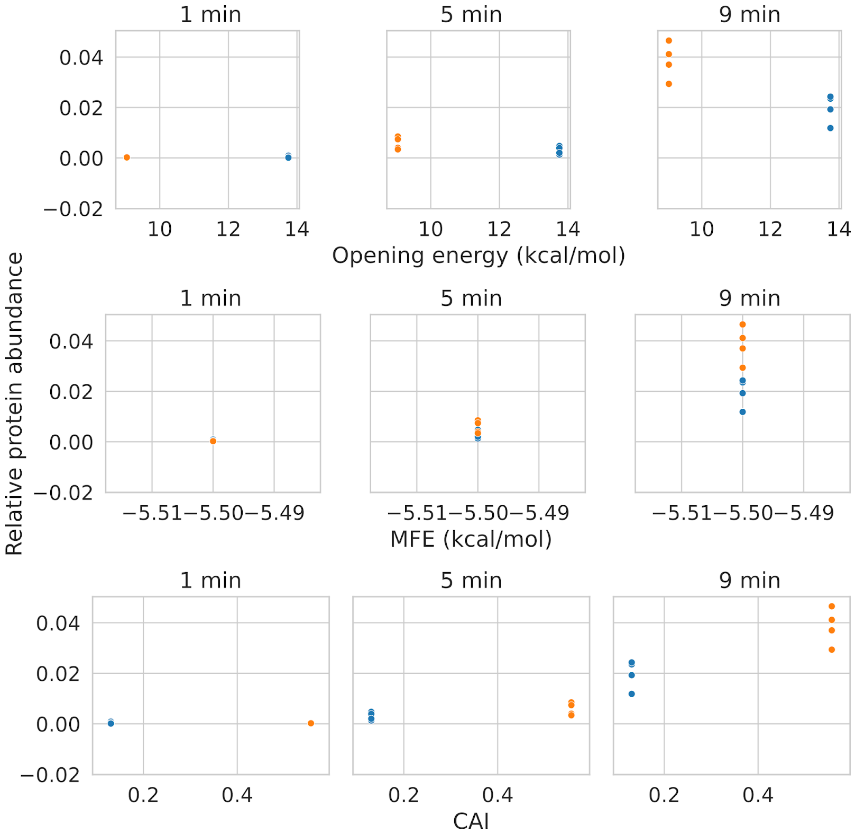

Figure R6. Accessibility is able to capture the full ensemble average energy of a sequence. The levels of major capsid protein expressed by the wild-type (orange) and mutant (blue) strains of bacteriophage T7 (Jack et al, 2017). The mutant major capsid gene was codon-deoptimised such that the first and the last 14 codons remained unchanged (Jack et al, 2017). Proteomic analyses were carried out at 1, 5 and 9 min post-infection in four biological replicates (Jack et al, 2017). Opening energies −24:24, MFEs −30:30, and CAI of the wild-type and mutant sequences were compared. The approximated 'Expression Scores' of the wild-type and mutant sequences are 89 and 38, respectively (opening energies of 9.05 kcal/mol and 13.76 kcal/mol, respectively). MFE, Minimum Free Energy; CAI, Codon Adaptation Index. Taken from the current version of our manuscript, S8 Figure.

Regarding 'MFE is usually calculated for a certain region and is a single value', the examples of input and output are given in the RNAfold manuals. To run RNAfold, the default input is a single sequence and the output is a single MFE value. See https://www.tbi.univie.ac.at/RNA/RNAfold.1.html#heading5

This applies to other tools that calculate MFE, e.g. UNAfold and NUPACK.

Here is an example using the tools RNAplfold and RNAfold to compute the opening energy and MFE. For a given sequence, MFE from RNAfold is a single value, whereas opening energy from RNAfold is a matrix of base pairing or equivalently energies for all possible subsegments. This can be readily seen by using the tools on an example sequence for eg: GFP,

ATGAAGAAGAGTTTGAGTGTGTCGGGGCCAGGGATGAAAGGGATGGTCGGCATGGCG
GCGCGCGTCTTTGCAGCGATGTCACGCGCCCGTATTTCCGTGGTGCTGATTACGCAATC
ATCTTCCGAATACAGCATCAGTTTCTGCGTTCCACAAAGCGACTGTGTGCGAGCTGAAC
GGGCAATGCAGGAAGAGTTCTACCTGGAAACCTCTGAGGATCATAAACACAAGGAGGAA

GATCATGACGGAGCTCGCCTCGAGGGAGGACTATCTATCTATCTATCTATCTTCGGCGGA
CGGACTACCATCGCATTACGGGGCTACGACGGACTCGATCTACTATCTATCTACTTCTAG
For this sequence, RNAfold gives the MFE of -111.00 kcal/mol, whereas RNAplfold (-W 210 -u 210 -O) gives a table of energies for different subsegments. A truncated part of that table is shown below:

#i$	l=1	2	3	4	5
1	0.03161493	NA	NA	NA	NA
2	0.03388246	0.05027386	NA	NA	NA
3	0.184498	0.2132506	0.2277539	NA	NA
4	0.1202859	0.1870739	0.2158873	0.2304196	NA

....

However, global folding (MFE using full length sequence) such as done above is a less common practise, unless the sequence is short enough. Instead, some regions are used for example, 1:30 by slicing the sequence from 1 to 30 and using it as input. But for RNAplfold, input of full sequence automatically gives energy for all regions and we need to figure out the regions with biologically relevant signals.

About my question as to why the region of the mRNA used for calculation is so different in different organisms, the authors say that it is because the SD region is absent in other eukaryotes. That can change the length of the sequence upstream of AUG, but why later?

See our detailed response to Reviewer 2 (concerning point 3 and 4).

Despite this, I think TIsigner is a useful program to optimize expression by modification of synonymous codons, but I don't really see much beyond that.

We agree that the TIsigner web server is a major outcome of this work. We used both computational analysis and experiments to demonstrate how to make use of our findings in recombinant protein production. These include the analyses of multiple, independent large-scale datasets, and our own experiments. Besides, we think that our stochastic simulation model is unique. To emphasise these points, we have added the following sentences in the main text to illustrate the contributions of this study:

> "The existing algorithms for gene optimisation sample synonymous protein-coding sequences use models based on CAI, tAI, MFE, and/or G+C content (%) [23–27]. However, these models are usually based on some of the above findings that rely on either endogenous proteins, reporter proteins, or a few heterologous proteins with their synonymous variants. It is unclear whether these features are generalisable to explain the expression of all heterologous proteins. To address this question, we studied multiple large-scale non-redundant recombinant protein production (N=11,430), proteomic

(N=3,725), and fluorescent reporter (N=82,002) datasets from bacterial and eukaryotic species in order to identify mRNA features that best explain variation in protein abundance. This problem has not previously been investigated with this scale of heterogeneous datasets in any previous studies.

We find that mRNA accessibility is a single best predictor of protein expression across the datasets, and accurately predicts the success or failure of 11,430 experiments of recombinant protein expression in *E. coli*. Specifically, the accessibility of translation initiation sites outperform other mRNA features by capturing all possible optimal or suboptimal structures beyond translation initiation sites. With this information, we propose how accessibility can be exploited to fine-tune recombinant protein expression at a low cost. Specifically, we built a web server called TIsigner (Translation Initiation coding region designer), which optimises a protein-coding sequence by suggesting synonymous codon changes within the first nine codons. Therefore, our approach makes gene optimisation accessible, as PCR can be used rather than an expensive full-length gene synthesis."
